# Cytokines and Chemokines in Breastmilk of SARS-CoV-2 Infected or COVID-19 Vaccinated Mothers

**DOI:** 10.3390/vaccines10122001

**Published:** 2022-11-24

**Authors:** Felicia Trofin, Olivia Simona Dorneanu, Daniela Constantinescu, Eduard Vasile Nastase, Cătălina Luncă, Luminița Smaranda Iancu, Ioana-Maria Andrioaie, Alexandru Duhaniuc, Corina Maria Cianga, Mariana Pavel-Tanasa, Dana-Teodora Anton-Păduraru, Petru Cianga

**Affiliations:** 1 Microbiology Department, University of Medicine and Pharmacy “Grigore T. Popa”, 700115 Iasi, Romania; 2 Clinical Hospital of Infectious Diseases “Sf. Parascheva”, 700116 Iasi, Romania; 3 Immunology Department, University of Medicine and Pharmacy “Grigore T. Popa”, 700115 Iasi, Romania; 4 Immunology Laboratory, “Sf. Spiridon” Clinical Hospital, 700111 Iasi, Romania; 5 Infectious Diseases Department, University of Medicine and Pharmacy “Grigore T. Popa”, 700115 Iasi, Romania; 6 ”Sf. Maria” Children Emergency Hospital, 700309 Iasi, Romania; 7 National Institute of Public Health, Iasi Regional Center for Public Health, 700465 Iasi, Romania; 8 Mother and Child Medicine Department, University of Medicine and Pharmacy “Grigore T. Popa”, 700115 Iasi, Romania

**Keywords:** cytokines, SARS-CoV-2, COVID-19, vaccine, breastmilk, antibodies

## Abstract

Introduction: The COVID-19 disease and anti-SARS-CoV-2 vaccination were accompanied by alterations in several inflammatory markers. The aim of our research was to check to what extent such cytokines are transferred to infants via the breastmilk of SARS-CoV-2-infected or vaccinated mothers. Thus, we wanted to check if breastfeeding is safe during SARS-CoV-2 infection or after COVID-19 mRNA-vaccination. Material and method: The Luminex Multiplexing Assay was used for quantifying 10 cytokine in the human breastmilk of SARS-CoV-2-infected or COVID-19-vaccinated mothers, compared with anti-SARS-CoV-2 IgG naïve mothers. Two milk samples were collected at 30 and 60 days either after the booster dose or afterthe onset of symptoms. A single milk sample was collected from the mothers within the control group. Results: The cytokine concentrations were mostly found within the reference intervals for all mothers. The status of the vaccinated/infected mother, the age of the breastfed child, the parity of the mother and the maternal age were variation factors of the above-mentioned cytokine concentrations. The type of birth and the presence of IgG in the milk had no influence on these cytokine concentrations in milk. Furthermore, no statistically significant differences were recorded between the cytokine concentrations of the two milk samples. Conclusion: Our study provides data that support the safety of breastfeeding in the case of mild COVID-19 infection or after Pfizer or Moderna vaccinations.

## 1. Introduction

The manifestations of SARS-CoV-2 infection can vary from asymptomatic or mild to critical and even fatal forms [1]. Thus far, there are several biological parameters that have been described to predict the fulminant evolution of the disease, such as increased C-reactive proteins (CRPs), lactate dehydrogenases (LDH) [2], the neutrophil/lymphocyte ratio (NLR), D-dimers and ferritin [3]. Some authors showed that the infected patient’s response to SARS-CoV-2 is embodied by an innate immune response followed by neutrophilia, lymphocytes apoptosis, a decrease in T cell numbers and an increase in some specific pro-inflammatory cytokine concentrations [4]. The hyper-inflammatory reaction, known as the cytokine release syndrome or cytokine storm, occurs due to the fulminant release of mostly inflammatory cytokines and chemokines [5,6,7,8,9]. Consequently, the high concentrations of some pro-inflammatory factors were highlighted as pathogenic factors in COVID-19: interleukin 6 (IL-6) [10]; IL-10 [11], IL-1 [12], IL-1β, IL-2, IL-4 and IL-5 [13]; IL-7, IL-12, tumor necrosis factor (TNF), interferon-gamma (INF-γ) and IFN-β [11]; colony-stimulating factors (CSF) and macrophage inflammatory protein 1 alpha (MIP1α) [10]; MIP-1B [6]. High levels of chemokines were also detected in severe forms of COVID-19: IFN-induced proteins-10 (IP-10/CXC-chemokine ligand 10 (CXCL10)), CXCL9, monocyte chemoattractant protein-1 (MCP1), chemokine ligand-2 (CCL)-2 and IL-8 [6]. TNF-α, produced by macrophages or monocytes during acute inflammation, is responsible for a diverse range of signaling events within cells, leading to necrosis or apoptosis [14]. IL-6 is promptly and transiently produced in response to infections and tissue injuries and contributes to host defense via the stimulation of acute phase responses, hematopoiesis and immune reactions [15]. IFN-γ, produced by CD4+ T helper type 1 lymphocytes, CD8+ T cytotoxic lymphocytes, NK cells, NKT cells and professional antigen-presenting cells, regulates innate and adaptive immunity to viral infection by cell growth inhibition and induced apoptosis. Granulocyte macrophage-CSF (GM-CSF), which is usually released by T cells at the same time as IFN-γ, is able to protect against the growth-inhibitory effects of IFN-γ on macrophages [16]. IL-10, an anti-inflammatory mediator, is a negative regulator of IFN-γ [16], ensuring the protection of the host from over-exuberant responses to pathogens [17]. IFN-β is secreted by macrophages and fibroblasts and elicits antiviral, anti-proliferative and immunomodulatory activities [18]. Bioactive IL-1β is a pro-inflammatory cytokine that is essential for the host response and resistance to pathogens; it also exacerbates damage during chronic disease and acute tissue injuries [19]. IL-2 promotes lymphopoiesis [16], contributes to optimal primary immune responses and has an essential role in developing efficient memory responses [20]. IP-10, also known as C-X-C motif chemokine 10, is an IFN-inducible protein produced by leukocytes, neutrophils, eosinophils, monocytes, epithelial, endothelial and stromal cells and keratinocytes in response to IFN-γ. It induces chemotaxis, apoptosis, cell growth inhibition and angiostasis [21]. IL-5 is produced by both hematopoietic and non-hematopoietic cells, including T cells, granulocytes and natural helper cells. It exerts effects for proliferation and differentiation [22].

One of the main roles of human milk is to protect the infant against infectious and inflammatory diseases. The World Health Organization (WHO) recommends breastfeeding to reduce child mortality [23]. Over time, the researchers evidenced the presence of various cytokines such as IL-1β [24]; IL-4 and IL-5 [25]; IL-6, IL-8, IL-10, IL-12 and TNF-α [26]; IL-13 [27]; IFN –γ [25]; G-CSF and GM -CSF [28,29]; M-CSF [30]; and IFN-α and IP-10 [31] in breastmilk in various maternal conditions. Narayanaswamy et al. (2022) detected a higher level of IFN-γ in the breastmilk of anti-SARS-CoV-2-vaccinated mothers 2–3 weeks after vaccination [32].

Considering the above-mentioned data and further taking into account that some blood molecules such as IgA and, to a much lesser extent, IgG are excreted in breastmilk following COVID-19 [33] or vaccinations both during pregnancy [34] or after birth [35], we aimed to check if the cytokines that are potentially released in COVID-19-affected mothers or after anti-SARS-CoV-2 vaccination are readily transferred to the breastfed child as well. Thus, we wanted to check if breastfeeding is safe during SARS-CoV-2 infection or after COVID-19 mRNA-vaccination. We hypothesized that cytokines may be transferred from a breastfeeding SARS-CoV-2-infected mother to her infant. Hence, the concentrations of the following cytokines in the breastmilk were measured by the Luminex Multiplexing Assay: TNF-α, IL-6, IFN-β, IL-10, IL-1β, IFN-γ, IL-2, GM-CSF, IL-5 and IP-10. We used the breastmilk of SARS-CoV-2-infected or anti-SARS-CoV-2-vaccinated mothers sampled at 30 and 60 days after the onset of infection or the second vaccine dose, respectively.

## 2. Materials and Methods

### 2.1. Study Design and Participants

The study was a prospective cohort study, and the aim was to detect the presence of 10 different cytokines in human breastmilk. The characteristics of the study groups are detailed in Supplementary Material Tables S1–S3. The inclusion criteria were as follows: (a) two doses of a mRNA vaccine; (b) no more than a month since the SARS-CoV-2 infection debut; and (c) anti-SARS-CoV-2 unvaccinated with no declared COVID-19 history (the control group). Based on these inclusion criteria, the participants were classified into three groups: COVID-19-vaccinated mothers, SARS-CoV-2-infected mothers and the control group. Mothers from the first 2 groups were vaccinated or infected during breastfeeding, and none were infected or vaccinated during pregnancy. We accepted mothers any time from the onset of breastfeeding, whether by direct breastfeeding or by pumped milk. All participants were volunteers, and they were enrolled after signing an informed consent form. Participants were asked for information regarding the following: the mother’s age, the date of child’s birth, parity, birth type, vaccination schedule, post-vaccination side effects, COVID-19 symptoms and signs and hospitalization.

### 2.2. Samples Collection

The anti-SARS-CoV-2-vaccinated and SARS-CoV-2-infected volunteers provided 2 human breastmilk samples: the first one was collected at precisely 30 days and the second one was collected at precisely 60 days after the second vaccine dose or after the onset of the symptoms. The mothers from the control group sampled the breastmilk as soon as possible to minimize the risk of a SARS-CoV-2 infection. The mothers collected their breastmilk by themselves by manual expression or breast pumping. Sampling was performed at home to ensure their personal comfort and familiar environment in order to maintain the usual composition of the milk and the evacuation rate. There were no restrictions in the mother and child’s everyday life since entering the study. Mothers were allowed to breastfeed between the 2 samplings. The participants received sampling kits accompanied by sampling and storage instructions. Breastmilk measuring 1 mL was collected in sterile tubes and expressed from the medium flow. The samples were immediately frozen at −20 °C and stored until further analyses. They were transported to the laboratory within no more than 30 min in refrigerated boxes so as to prevent thawing.

### 2.3. Analysis of Samples

The specimens were thawed and they were allowed to slowly reach room temperature. Then, they were vortexed for 20 s. For milk degreasing, we centrifuged the tubes at 5000× *g* for 25 min at 4 °C. The skimmed milk was transferred into a new tube for further processing.

All breastmilk samples were checked for the presence of reactive anti-S1 RBD IgG antibodies using a sandwich enzyme-linked immunosorbent assay (ELISA), as previously described in Trofin et al. (2022) [25]. We used a 1:10 dilution in the sample diluent provided by the kit. The samples were processed in accordance to the manufacturer’s indications (TestLine Clinical Diagnostics, Czech Republic, Catalog number: CoRG96-EIA COVID-19 RBD IgG, test specificity of 99.15% and test sensitivity of 99.9%). The optical densities were measured with a TECAN Infinite 200 photometer at 450 nm wavelength, and the results were processed with the Magellan software.

Ten cytokines and chemokines were simultaneously measured from the same breastmilk sample using a bead-based immunoassay with the Luminex Multiplexing Assay. We followed the manufacturer’s instructions (Human Premixed Multi-Analyte Kit, R&D systems, Minneapolis, MN, USA, Catalog number: LXSHM-10). The samples were analyzed using a Luminex 200 (USA) device. We quantified the following cytokines: TNF-α, IL-6, IFN-β, IL-10, IL-1β, IFN-γ, IL-2, GM-CSF, IL-5 and IP-10.

### 2.4. Ethical Principles

The study complied with the ethical principles stated by the World Medical Association’s Declaration of Helsinki regarding medical research involving human subjects. The study was approved by the Commission of Ethics of Research from the University of Medicine and Pharmacy “Grigore T. Popa” Iasi, Romania (IRB number: 211/2022).

### 2.5. Statistical Analysis

Statistical analysis was conducted using the 20th version of the IBM SPSS statistical software. The Kolmogorov–Smirnov test was used to assess the distribution of the variables. The correlations between the variables were verified using Pearson or Spearman’s correlation tests. The *p*-value was used to assess the α-significance level. A significance level under 0.05 indicates a probability of less than 5% that the event happened by chance. The α-significance level highlights the probability of false positives. The correlation ranking was established using the r results as follows: 0–0.29—poor correlation; 0.3–0.49—medium correlation; 0.5–1—strong correlation. We compared all groups using the independent sample t-test or paired-samples t-test. ROC analysis (including AUC—area under curve values) was performed in order to assess the sensitivity and sensibility of the investigated biomarkers in predicting the infection status. The conclusions of our study were related to the data generated by the statistical analysis. The data were analyzed for the entire study group and also for the three individual subgroups.

## 3. Results

### 3.1. Characteristics of the Study Groups

The study was performed on 65 breastfeeding mothers. They were classified into three different groups depending on the inclusion criteria: 26 volunteers were anti-SARS-CoV-2 vaccinated, with no history of COVID-19 until sampling, 22 volunteers were recently infected by SARS-CoV-2, and the control group consisted of 17 anti-SARS-CoV-2 unvaccinated mothers, without any known history of COVID-19. The control group initially consisted of 17 lactating mothers who declaratively did not have any specific symptoms of COVID-19 from 2020 to present days, implicitly did not have positive rapid antigen tests or RT-PCR tests and were not vaccinated with any of the developed anti-SARS-CoV-2 vaccines. In order to confirm the accuracy of the control group and that these mothers did not experience an asymptomatic form of COVID-19, we tested for the presence of anti-SARS-CoV-2 IgG in their breastmilk. Seven milk samples from the control group were positive for anti-SARS-CoV-2 IgG; hence, we decided to exclude them from this group. The final control group consisted of 10 anti-SARS-CoV-2 IgG-negative milk samples. The maternal age ranged from 29 to 44 years old and the breastfed infants age ranged between 2 and 34 months. They lived in urban areas and they were working in different fields (data regarding the study groups are available in Supplementary Materials).

#### 3.1.1. The Group of Vaccinated Mothers

The mean age of vaccinated mothers and of their breastfed infants was 33.2 (standard deviation (σ) = 2.44) years old and 13.9 (σ = 10.21) months old, respectively. Twenty-three (88.5%) mothers were vaccinated using the Pfizer-BioNTech vaccine and three (11.5%) were vaccinated with the Moderna vaccine. Seven (26.9%) infants were born by natural birth and nineteen (73.1%) experienced cesarean birth. For 17 (65.4%) women, the breastfed baby was their first born; for 8 (30.8%) women, the baby was the second child; for 1 (3.8%) women, the baby was the third child. Seventeen (65.4%) volunteers described mild side effects after vaccination, four (15.4%) reported medium side effects and five (19.2%) reported no side effects (Supplementary Materials—Table S1. Characteristics of the anti-SARS-CoV-2-vaccinated mother group).

#### 3.1.2. The Group of COVID-19 Mothers

The mean age of these mothers and of their breastfed infants was 32.95 (σ = 2.93) years old and 13 (σ = 8.56) months old, respectively. Eight (36.4%) infants were born by natural birth and 14 (63.6%) were born by cesarean birth. Eight (36.4%) mothers were primiparous and fourteen (63,6%) of them were multiparous. Sixteen (72.7%) mothers were vaccinated using two doses of a mRNA vaccine prior to SARS-CoV-2 infection, and the other six (27.3%) mothers did not receive any dose of any COVID-19 vaccine. None were hospitalized (Supplementary Materials—Table S2. Characteristics of the SARS-CoV-2-infected mother group).

#### 3.1.3. The Control Group

The control group included 10 anti-SARS-CoV-2 IgG-negative breastmilk samples. The mean ages were 32.1 (σ = 2.13) years old and 10 (σ = 6.62) months old for the mothers and their newborns, respectively. Four (40%) infants were born naturally and six (60%) were born by cesarean section. Two (20%) infants were firstborns, seven (70%) were second-born and one (10%) was third-born in their family (Supplementary Materials—Table S3. Characteristics of the control group).

### 3.2. Cytokine Assessment

TNF-α, IL-6, IFN-β, IL-10, IL-1β, IFN-γ, IL-2, GM-CSF, IL-5 and IP-10 were tested using the Luminex Multiplexing Assay. Most cytokine concentrations were found within the reference intervals in the mothers’ milk of all three groups. The relevant statistical parameters (mean, σ, median and inter quartile range values) are listed for all investigated cytokines in Table 1 and Table 2. TNF-α levels were elevated above the reference threshold in one (3.85%) vaccinated mother and five (22.7%) infected ones. No volunteers from the control group had elevated values. However, the mean TNF-α value in the vaccinated group was similar with the mean value within the control group, and they both were about 5 times smaller than the mean value in the infected group. Six (23.1%) vaccinated mothers, six (27.3%) infected mothers and three (30%) mothers from the control group showed higher values during the IL-6 test. The mean IL-6 concentration in the control group was 5 times lower than the mean IL-6 in the group of vaccinated women, which in turn was 5 times lower than the mean IL-6 in the group of infected women. Five (19.2%) vaccinated volunteers and five infected mothers (22.7%) had slightly augmented concentrations of IL-10. Four (15.4%) and five (22.7%) subjects from the vaccinated and infected group, respectively, showed higher levels of IL-1β. The mean IL-1β value in the infected group was about 10 times higher than the mean IL-1β in the vaccinated or control group. IL-2 was elevated in only one (4.5%) infected mother, the mean value being 5 times higher in the group of infected persons than in the others. GM-CSF was augmented in two (7.7%) vaccinated and in seven (31.8%) infected participants, the mean GM-CSF levels being raised 1.5 times in the group of infected people compared to the others. All mothers excreted IP-10 in high concentrations in breastmilk, with no considerable differences between the three groups. None of the three groups had high values for IFN-β, IFN-γ or IL-5 (Table 3). Anti-S1 RBD IgGs were detected in all SARS-CoV-2-infected or -vaccinated mothers’ breastmilk. Data regarding the study group cytokine concentrations are included in Supplementary Materials Figures S1–S18.

### 3.3. Statistical Analysis

The cytokine concentrations were correlated with each other, showing medium or strong relations. The values of TNF-α, IL-2 and IFN-β and IL-5 from the second sampling were moderately correlated with the affiliation to a certain group: vaccinated, SARS-CoV-2-infected or the control (Table 4). The infants’ age was moderately directly correlated with the IL-6 and IP-10 concentrations from the second sampling, but the newborns’ rank was moderately inversely correlated with the GM-CSF and IP-10 from the first sampling (Table 4). The TNF-α value was inversely correlated with the mothers’ age, which is poorly related (Table 4). The type of birth is not correlated with any other parameter (*p* > 0.05).

For group statistics, we used the independent sample test to compare the cytokine results depending on the prior vaccination, type of birth, child’s rank and IgG presence in breastmilk. There were no significant differences registered (*p* > 0.05). No significant differences were registered between the cytokines concentrations among the three groups according to the one-way ANOVA test (*p* > 0.05). Moreover, no significant differences were observed between the two samplings, according to paired samples test (*p* > 0.05).

#### 3.3.1. Statistical Analysis of the Vaccinated Mothers Group

The variables of these cytokine results were directly correlated with each other. In this group, the child’s age directly correlates with GM-CSF, IP-10 values from the first sampling and TNF-α, IL-6, IFN-β and IP-10 concentrations from the second sampling (Table 5). The mother’s parity correlation with GM-CSF, IP-10 and TNF-α from both samplings and IL-10 from the second sampling is inverse (Table 5). Medium direct correlations were registered between the type of birth and IL-1 and IL-5 breastmilk levels (Table 5). The mothers’ age and the type of the side effects after vaccination did not correlate with any other parameter (*p* > 0.05).

The independent sample test did not show significant differences between the cytokine concentrations depending on the infant’s rank, the type of birth and the side effects after COVID-19 vaccination (*p* > 0.05). The paired samples test did not show significant differences between the first sampling and the second sampling results (*p* > 0.05).

#### 3.3.2. Statistical Analysis for the Group of Infected Mothers

The cytokine concentrations were directly correlated with each other, possessing a medium or strong connection. The infant and the mother’s age, the infant’s rank, the type of birth, the symptoms and the vaccination prior to the COVID-19 disease did not correlate with cytokine concentrations (*p* > 0.05). There were no significant differences between the cytokines results depending on the child’s rank, the type of birth, the anti-SARS-CoV-2 vaccination prior COVID-19 or the COVID-19 symptoms (*p* > 0.05) (Table 6). Moreover, there were no significant differences between the first sampling and the second sampling results (*p* > 0.05).

#### 3.3.3. Statistical Analysis for the Control Group

The cytokines’ concentrations were directly correlated with each other, possessing a medium or strong connection. The infant and the mother’s age, the infant’s rank and the type of birth did not correlate with cytokine concentrations (*p* > 0.05). There were no significant differences between the cytokines’ results depending on the infant’s rank or birth type (*p* > 0.05).

To further assess the sensitivity and sensibility of the investigated biomarkers in predicting the infection status or the vaccination response, we performed several ROC analyses. The variables that best predicted the infection status were the TNF-alpha and IL-2 value from the second sampling, leading to AUC values of 0.742 and 0.745, respectively (Supplementary Materials Figure S19). The corresponding cut-off values were 1 pg/mL (sensitivity = 0.857; specificity = 0.577) for TNF-α and 3.4 pg/mL (sensitivity = 0.810; specificity = 0.692) for IL-2. However, the combined model of two variables did not lead to an improved analysis.

## 4. Discussion

It is well documented that for COVID-19 infections, the levels of different pro-inflammatory cytokines can increase steeply, sometimes culminating in a “cytokine storm” [2,3,4,5,6,7,8,9]. We were interested in examining the response of the organism against SARS-CoV-2, as well as the response against the vaccine stimuli reflected in human breastmilk. A growing number of soluble factors have been identified in this secretion and it is still unclear if these factors are excreted or secreted by the glandular epithelial cells [36]. It is most likely that both mechanisms are in place and responsible for the presence of a soluble factor or another factor in milk, as it may well be that some cytokines take advantage of both mechanisms in order to be present in milk secretions. However, if we consider data published by Narayanaswamy et al. (2022) [32] showing an increase in the IFNγ breastmilk concentration soon after vaccination, as this is an immune interferon, then there is a higher probability that this cytokine is transferred from blood to milk, and the milk concentration mirrors the blood concentration. If so, this mechanism of excretion could be extended to many other cytokines.

Human breastmilk is the basic and gold standard food for newborns during the first year of life. They receive both nutrients and immunity fragments such as immunomodulatory, anti-inflammatory or antimicrobial fragments by ingesting human milk [37,38,39]. The literature’s data regarding the effect of breastfeeding on health outcomes mention that breastfed infants are at a significantly lower risk for some infections, especially respiratory tract infections, gastroenteritis, otitis, type 2 diabetes mellitus [37], obesity, allergy and celiac disease compared to non-breastfed children [38]. Other studies show that breastfeeding reduces morbidity risks or even of sudden infant death syndrome [37]. Breastmilk boosts brain developments, favoring white matter and resulting in an improvement in intelligence [37]. On the other hand, breastmilk benefits are much broader, taking into account the child’s emotional, cognitive, psychomotor, psychological, socioeconomic and environmental development [38,39]. Other authors suggest that various cytokines that are normally observed in small amounts in human breastmilk can directly modulate the immunological development of the recipient infant [24]. Garofalo (2010) suggested that anti-inflammatory cytokines, chemoattractants and activators have roles in compensating the developmental delay of the neonate immune system [40].

However, all these benefits must be subjected to a cost–benefit balance at certain times. How should one proceed when the child can be at risk due to the transfer of harmful substances via milk. In this idea, we set out to demonstrate that breastmilk remains safe for infant feeding after a mild SARS-CoV-2 infection and, even more so, after anti-SARS-CoV-2 vaccinations.

To the best of our knowledge, there are no studies that aimed to detect or quantify the presence of cytokines in human milk after COVID-19 and only one tested 10 cytokines across 3 weeks after SARS-CoV-2 vaccinations [32].

We included in our tests a set of cytokines and chemokines with serum concentrations that were shown to increase during COVID-19 [1,2,3,4,5,6,7,8,9,41,42], and this increase might also be reflected in breastmilk: TNF-α, IL-6, IFN-β, IL-1β, IFN-γ, IL-2, GM-CSF and IL-5, IP-10. We were also interested in the immunoregulatory and antifibrotic functions of IL-10. The clinical significance of highly elevated IL-10 amounts in the serum of COVID-19 patients has been generally regarded as an anti-inflammatory or immune-inhibitory mechanism [43].

The first step in our study was to detect the anti-RBD IgG type in all breastmilk specimens. In all human milk samples from the vaccinated and COVID-19 lactating women, anti-SARS-CoV-2 IgG was detected, thus showing that these organisms were responsive towards this antigen. These results are in agreement with our previous data where we also highlighted the presence of anti-S1 RBD IgG in the breastmilk of anti-SARS-CoV-2-vaccinated mothers [35]. This is also in line with many other study results [16,25].

The group of SARS-CoV-2-infected mothers included 16 anti-SARS-CoV-2-vaccinated mothers prior to infection, while 6 were unvaccinated prior to infection. Vaccinated and infected mothers could have constituted a distinctive group due to the triple immunization (COVID-19 and two anti-COVID-19 vaccine doses). Since only six mothers were not vaccinated before COVID-19, we were unable to include them in a separate group due to the insufficient size of this group that would not have yielded statistically significant results. Although we expected enhanced responses in terms of cytokine milk transfers in mothers vaccinated before infection, the differences recorded between the two subgroups were not statistically significant.

In most of the samples, the concentrations of the tested cytokines were below the upper limit of the reference interval. However, we found that the concentrations of several cytokines were elevated in the breastmilk of some infected subjects. TNF-α increased in 22.7% of the infected mothers, resulting a mean value that is 5 times higher than the mean of the vaccinated or control groups. Interestingly, higher values of IL-6 were detected in all three groups; however, the average concentration per group was different. For instance, the mean IL-6 concentration value of COVID-19 mothers was 5 times higher than that of the vaccinated group, which in turn was five times higher than the control group.

For IL-5, IFN-β and IFN-γ, the concentrations were within the reference interval in the entire group, with this last result being different from the ones published by Narayanaswamy et al. (2022) [32]. Five (19.2%) vaccinated volunteers and five infected mothers (22.7%) had slightly elevated concentrations of IL-10. At this point, we could speculate that these results may be also due to a potential increase in IL-6 and IL-10 levels in the serum of previously infected SARS-CoV-2 females compared to the infection-naive group, as it was observed in a previous study [41]. The mean of IL-1β values for the infected subjects was 10 times higher than for vaccinated ones. This difference came from the high values recorded in five lactating and infected volunteers. Although the mean IL-2 was 5 times higher in infected mothers compared to the others, IL-2 concentrations did not exceed the upper reference value. Elevated GM-CSF concentrations were recorded in the highest number of cases; however, this was observed exclusively within the infected mothers group. Even so, the mean GM-CSF values were not much higher in post-COVID-19 milk compared to the reference interval. The IFN-inducible protein, IP-10, was found in high concentrations in the milk of all three groups. Our findings are in line with Burch et al. (2013) [44], who concluded that IP-10 showed higher concentrations than other cytokines (mean IP-10 concentration 395 pg/mL (Burch et al. (2013) vs. 270.25 pg/mL (our findings)) in breastmilk.

IFN-β is not secreted in breastmilk. Hale et al. (2012) suggested that interferon β-1a does not penetrate the milk compartment significantly in women receiving interferon β-1a [45]. Our findings are supported by Hale et al.’s (2012) study who demonstrated that, even after intramuscular IFN-β-1a administration, the baby is not exposed to IFN-β-1a via breastmilk.

Our results are similar with breastmilk cytokine concentrations found by Dawod and Marshall (2019) [46], as provided in Table 1.

Even if there are some differences between the cytokine concentrations within the 3 groups, according to the one-way ANOVA test, the differences were not statistically significant (*p* > 0.05). Moreover, when using the paired samples test, it became evident that the cytokines’ milk concentrations from the second sampling did not diverge from those of the first sampling (*p* > 0.05).

However, the affiliation to the group of vaccinated or infected mothers has a medium correlation with higher values of TNF-α, IL-2 and IFN-β and IL-5 from the second sampling (Table 2). The results of Spearman’s test outline the increase in TNF-α and IL-2 concentrations in vaccinated and infected subjects (Table 2). The cytokine concentrations were directly correlated to each other, with their concentrations being dependent one of each other.

The concentration of IL-6 and IP-10 in the milk of the second sampling increased with the age of the breastfed child (Table 2). GM-CSF and IP-10 concentrations from the first sampling decrease when the mother’s parity increased (Table 2). TNF-α concentrations in breastmilk decreased with the increasing age of the mother (Table 2). The type of birth does not seem to influence the concentrations of cytokines in breastmilk (*p* > 0.05). No significant differences were recorded between the cytokine concentrations depending on the anti-SARS-CoV-2 vaccination in the antecedents, the type of birth, the parity of the mother and the presence of anti-SARS-CoV-2 IgG in milk (*p* > 0.05).

In the group of anti-SARS-CoV-2-vaccinated mothers, GM-CSF and IP-10 from the first sampling and TNF-α, IL-6, IFN-β and IP-10 from the second sampling increased as the child’s age increased (Table 3). GM-CSF, IP-10 and TNF-α concentrations from both samplings and IL-10 from the second sampling decreased as the parity of the mother increased (Table 3). IL-1 and IL-5 concentrations were elevated in mothers who had a cesarean section birth (Table 3). The mothers’ age and the occurrence of the side effects after vaccination did not influence cytokine concentrations (*p* > 0.05). No significant differences were observed between cytokine concentrations depending on the infant’s rank, the type of birth or the side effects of the COVID-19 vaccination (*p* > 0.05). There were no significant differences between the first sampling and the second sampling’s results (*p* > 0.05).

Narayanaswamy et al. (2022) [32] measured cytokine concentrations in the breastmilk of anti-SARS-CoV-2-vaccinated mothers who reported side effects such as fever, fatigability, local pain or headaches. They observed that IFN-γ significantly increases after the first dose and then after the second vaccine dose. Women who reported any side effects had significantly higher levels of IFN-γ. However, the levels of IL-2, IL-6, IL-8, IL-10, IL-13, IL-1β and TNF-α did not differ across vaccinated and unvaccinated mothers. In our study, the concentrations of IFN-γ did not increase, and they were not influenced by the occurrence of reported vaccine side effects, such as fatigue, local pain or flu-like symptoms. This fact may be due to the timing of the samplings since Narayanaswamy et al. (2022) [32] chose to sample the breastmilk immediately after vaccination, while we sampled breastmilk at 30 and 60 days after the second dose. Narayanaswamy et al. (2022) [32] and us found that IL-2, IL-6, IL-10, IL-1β and TNF-α did not differ significantly in vaccinated mothers compared to the control group.

In the group of infected mothers, cytokine concentrations were not influenced by the infant’s and the mother’s age, the mother’s parity, the type of birth, the disease symptoms and the anti-SARS-CoV-2 vaccination prior to the infection (*p* > 0.05). There were no significant differences between cytokine concentrations depending on the mother’s parity, the type of birth, the anti-SARS-CoV-2 vaccination prior to COVID-19 or COVID-19 symptoms (*p* > 0.05). Moreover, there were not significant differences between the results of the two samplings (*p* > 0.05). However, the TNF-alpha and IL-2 values from the second sampling were generally higher in the infected group, as revealed by the ROC analysis, probably suggesting a more pronounced Th1 response.

The group of anti-SARS-CoV-2 unvaccinated, non-infected mothers showed results that did not correlate significantly with the infant and mother’s age, the infant’s rank or the type of birth (*p* > 0.05). There were no significant differences between the cytokine results depending on the infant’s rank or birth type (*p* > 0.05).

Overall, the mechanisms by which human cytokines reach into breastmilk remain elusive. We can only speculate that they can be excreted and their milk concentrations mirror, to a certain point, the serum ones. Cytokines triggered by either SARS-CoV-2 in mild COVID-19-affected patients or vaccinated volunteerswere not transferred in high concentrations to infants via human breastmilk 30 and 60 days after the onset of the disease, respectively. TNF-α, IL-6, IFN-β, IL-10, IL-1β, IFN-γ, IL-2, GM-CSF and IL-5 concentrations were in the reference range in the breastmilk of anti-SARS-CoV-2-vaccinated or SARS-CoV-2-infected mothers. IP-10 is secreted in the breastmilk of all mothers in high concentrations. TNF-α and IL-2 levels were higher in infected subjects than in vaccinated ones or in the control group, but they are still within the reference interval. The vaccine, the manifest disease, the age of the breastfed child, the mother’s parity and maternal age contributed to the variation. The type of birth had no impact on cytokine concentrations. Anti-S1 RBD IgG is secreted for at least 60 days in both SARS-CoV-2-infected or vaccinated mothers. The cytokine concentrations of infected and previously vaccinated mothers did not significantly increase compared to those of the unvaccinated and infected mothers.

Our study has several shortcomings. We were not able to assess the sera concentrations of the cytokines as the mothers consented only to milk sampling. The correlation of serum levels with those of breastmilk would have been very useful to obtain a better insight of the transfer mechanism. Samples collected closer to the day of vaccination or the onset of the COVID-19 would probably have generated more accurate results, but our motivation in setting the timing consisted in choosing a fixed, constant sampling period, which could be respected by all participants in the study. If we had chosen a period when the infection was still active, sampling would have been difficult due to the general altered state of the sick mothers. In such cases, should the babies present signs of disease, this would have shifted the mothers’ focus from the sampling of milk to the newborn. Therefore, we preferred to wait until the mothers and their babies returned to healthier states.

Our ascertainments encourage breastfeeding even after COVID-19 or anti-SARS-CoV-2 vaccinations. The World Health Organization [47], the Academy of Breastfeeding Medicine [48], the United Nations International Children’s Emergency Fund [49], the Society for Maternal-Fetal Medicine [50] and Centers for Disease Control and Prevention [51] emboldened breastfeeding during the COVID-19 pandemic or after vaccination. The previous statement is supported by the benefits that breastfeeding has on the health, immunity and psychosomatic development of the infant as well as on the mother both in short- and long-term periods.

## 5. Conclusions

Our study supports with scientific evidence the safety of breastfeeding in cases of mild COVID- 19 infection or vaccination. However, breastfeeding with severe COVID-19 should be further investigated. Therefore, we combine the official recommendations with scientific data and suggest not interrupting breastfeeding in cases of mild COVID-19 or after anti-SARS-CoV-2 vaccination. We also demonstrate the advantages of breastfeeding by highlighting that anti-SARS-CoV-2 antibodies are transferred after COVID-19 disease or vaccination.

## Figures and Tables

**Table 1:** Comparison of the cytokine concentrations excreted in human breastmilk as found in the literature with the reference values of the test kit and the range of concentrations, mean concentration, standard deviation and median obtained in the present study.

Cytokine	Concentrations of Cytokines in Breastmilk (pg/mL)	Blood Reference Concentrations (Luminex Kit Insert) (pg/mL)	Range of Concentrations in Our Assay (pg/mL)	Mean Concentration in Our Assay (pg/mL)	Standard Deviation in Our Assay (pg/mL)	Median Concentration in Our Assay (pg/mL)	Notes for the Present Study
IFN-γ	0.7–175	<40.33	0.8–40.33	7.48	4.32	5.99	All samples were <40.33 pg/mL.
IL-1β	0.028–23	<14.81	0.06–1836	33.89	184.5	5.82	84.5% of all samples were < 14.81 pg/mL; 1836 pg/mL was an extreme, isolated value recorded in one of the infected mothers.
IL-5	6.2–142	<7.08	<7.08	1.54	0.79	1.52	All samples were <7.08 pg/mL.
IL-6	3.5–148.6	<3.7	0.21–2087	38.62	225.35	0.95	93.1% of all samples were <142 pg/mL. 74.1% of all samples were <3.7 pg/mL. 2087 pg/mL was an extreme, isolated concentration recorded in the same sample as the extreme IL-1β.
IL-10	0–246	<4.2	0.8–8.7	2.18	1.28	1.79	86.2% of all samples were <4.2 pg/mL.
TNF	4.4–58	<6.85 (TNF-α)	0.02–168 (TNF-α)	5.05 (TNF-α)	21.81 (TNF-α)	1.07 (TNF-α)	89.7% of all samples were <6.85 pg/mL.
GM-CSF	1.6	<12.72	0.2–180.4	9.31	21.5	4.78	84.5% of all samples were <12.72 pg/mL.
IFN-β		<16.05	2.84–20.65	3.87	1.73	3.65	
IL-2		<31.65	2.46–429.46	11.26	52.56	3.69	
IP-10		3.50	7.37–1948.83	395.79	455.38	194.01	

**Table 2:** Interquartile range of the cytokine concentrations excreted in human breastmilk of all tested subjects.

		TNF-α (pg/mL)	IL-6 (pg/mL)	IFN-β (pg/mL)	IL-10 (pg/mL)	IL-1β (pg/mL)	IFN-γ (pg/mL)	IL-2 (pg/mL)	GM-CSF (pg/mL)	IL-5 (pg/mL)	IP-10 (pg/mL)
**Percentiles**	**25**	0.7614	0.6518	3.2402	1.5047	4.6550	5.3323	3.2787	3.5425	1.3034	73.2166
	**50**	1.0659	0.9547	3.6452	1.7941	5.8187	5.9988	3.6885	4.7809	1.5207	194.0144
	**75**	1.3704	3.1942	4.0502	2.2281	7.1922	7.9984	4.0983	8.2413	1.5207	544.2318

**Table 3:** The number (and percentage) of mothers with elevated concentrations of cytokines corresponding to each study group.

Tested Cytokines/Study Groups	*The Group of Vaccinated Mothers*	*The Group of COVID-19 Mothers*	*The Control Group*
**TNF-α**	1 (3.85%)	5 (22.7%)	0
**IL-6**	6 (23.1%)	6 (27.3%)	3 (30%)
**IL-10**	5 (19.2%)	5 (22.7%)	0
**IL-1β**	4 (15.4%)	5 (22.7%)	0
**IL-2**	0	1	0
**GM-CSF**	2 (7.7%)	7 (31.8%)	0
**IP-10**	26 (100%)	22 (100%)	10 (100%)
**IFN-β**	0	0	0
**IFN-γ**	0	0	0
**IL-5**	0	0	0

**Table 4:** *p* and *r* correlation coefficients related to the statistically significant correlations recorded in the entire group of subjects.

*Variables*	TNF-α	IL-2	IFN-β	IL-5	IL-6	GM-CSF	IP-10
**Affiliation to each characteristic group**	***p* = 0.005** ***r* = 0.361**	***p* = 0.022** ***r* = 0.301**	***p* = 0.031** ***r* = 0.314**	***p* = 0.010** ***r* = 0.372**	*p* = 0.114 *r* = 0.234	*p* = 0.650 *r* = 0.061	*p* = 0.670 *r* = 0.057
**Child’s age**	*p* = 0.991 *r* = −0.002	*p* = 0.932 *r* = 0.011	*p* = 0.167 *r* = 0.205	*p* = 0.979 *r* = 0.004	***p* = 0.017** ***r* = 0.347**	*p* = 0.148 *r* = 0.192	***p* = 0.015** ***r* = 0.354**
**Mother’s parity**	*p* = 0.411 *r* = −0.110	*p* = 0.719 *r* = −0.048	*p* = 0.731 *r* = −0.052	*p* = 0.620 *r* = −0.074	*p* = 0.415 *r* = −0.122	***p* = 0.015** ***r* = −0.318**	***p* = 0.042** ***r* = −0.267**
**Mother’s age**	***p* = 0.046** ***r* = −0.263**	*p* = 0.145 *r* = −0.194	*p* = 0.256 *r* = −0.169	*p* = 0.923 *r* = 0.015	*p* = 0.845 *r* = 0.029	*p* = 0.170 *r* = −0.183	*p* = 0.199 *r* = −0.171

*p* = coefficient of statistical significance; *r* = correlation coefficients.

**Table 5:** *p* and *r* correlation coefficients related to the statistically significant correlations recorded in the group of vaccinated subjects.

*Variables*	GM-CSF	IP-10	TNF-α	IL-6	IFN-β	IL-1β	IL-5
**Child’s age**	***p* = 0.030** ***r* = 0.427**	***p* = 0.005** ***r* = 0.536**	***p* = 0.012** ***r* = 0.484**	***p* = 0.010** ***r* = 0.495**	***p* = 0.036** ***r* = 0.414**	*p* = 0.353 *r* = 0.190	*p* = 0.996 *r* = 0.008
**Mother’s parity**	***p* = 0.002** ***r* = −0.572**	***p* = 0.001** ***r* = −0.609**	***p* = 0.017** ***r* = −0.464**	*p* = 0.090 *r* = −0.339	*p* = 0.096 *r* = −0.389	*p* = 0.712 *r* = −0.076	*p* = 0.189 *r* = −0.266
**Type of birth**	*p* = 0.397 *r* = 0.173	*p* = 0.351 *r* = 0.191	*p* = 0.188 *r* = 0.267	*p* = 0.343 *r* = 0.194	*p* = 0.102 *r* = 0.328	***p* = 0.039** ***r* = 0.407**	***p* = 0.027** ***r* = 0.434**

*p* = coefficient of statistical significance; *r* = correlation coefficients.

**Table 6:** *p*-value in Spearman’s correlation in the group of COVID-19 subjects.

	TNF-α	IL-6	IFN-β	IL-10	IL-1β	IFN-γ	IL-2	GM-CSF	IL-5	IP-10
**Child’s age**	0.420	0.973	0.705	0.633	0.550	0.376	0.605	0.690	0.226	0.992
**Mother’s parity**	0.973	0.974	0.435	0.921	0.920	0.947	0.710	0.644	0.731	0.948
**Mother’s age**	0.598	0.540	0.061	0.900	0.560	0.550	0.664	0.792	0.983	0.836
**Type of birth**	0.615	0.766	0.973	0.333	0.329	0.123	0.202	0.466	0.265	0.286
**Vaccination prior COVID-19**	0.562	0.278	0.662	0.543	0.102	1.000	0.397	0.593	0.824	0.943
**Symptoms**	0.922	0.295	0.843	0.897	0.845	0.491	0.550	0.898	0.544	0.872

## Data Availability

The data presented in this study are available in Supplementary Tables S1–S3.

## Supplementary Materials

The following supporting information can be downloaded at: https://www.mdpi.com/article/10.3390/vaccines10122001/s1: Table S1: Characteristics of anti-SARS-CoV-2 vaccinated mothers group, Table S2: Characteristics of SARS-CoV-2 infected mothers group, Table S3: Characteristics of the control group, Figure S1: TNF-α concentrations range according to each study group, Figure S2: TNF-α concentrations range according to each study group, without extreme outliners for a better visibility, Figure S3: IFN-β concentrations range according to each study group, Figure S4: IFN-β concentrations range according to each study group, without extreme outliners for a better visibility, Figure S5: IFN-γ concentrations range according to each study group, Figure S6: IL-1β concentrations range according to each study group, Figure S7: IL-1β concentrations range according to each study group, without extreme outliners for a better visibility, Figure S8: IL-2 concentrations range according to each study group, Figure S9: IL-2 concentrations range according to each study group, without extreme outliners for a better visibility, Figure S10: IL-5 concentrations range according to each study group, Figure S11: IL-5 concentrations range according to each study group, without extreme outliners for a better visibility, Figure S12: IL-6 concentrations range according to each study group, Figure S13: IL-6 concentrations range according to each study group, without extreme outliners for a better visibility, Figure S14: IL-10 concentrations range according to each study group, Figure S15: IL-10 concentrations range according to each study group, without extreme outliners for a better visibility, Figure S16: GM-CSF concentrations range according to each study group, Figure S17: GM-CSF concentrations range according to each study group, without extreme outliners for a better visibility, Figure S18: IP-10 concentrations range according to each study group, Figure S19: ROC curves generated for biomarkers that best predicted the infection status: TNF-alpha and IL-2.
