# Supplementary material for: Cytokines and Chemokines in Breastmilk of SARS-CoV-2 Infected or COVID-19 Vaccinated Mothers
Source: Vaccines (Basel). 2022 Nov 24;10(12):2001. doi: 10.3390/vaccines10122001 (PMC9784508; doi:10.3390/vaccines10122001)
Supplement: Supplementary file 1 [file vaccines-10-02001-s001.zip › vaccines-2036574-supplementary.pdf]

Supplementary Materials

Table S1. Characteristics of anti-SARS – CoV – 2 vaccinated mothers group

ID No	Anti-RBD IgG	Child`s age (months)	Mother`s age (years)	Mother`s parity	Birth type	Vaccine type	Side effects after vaccination
1	+	9	30	1	Cesarean section	Pfizer	Local pain
2	+	23	31	1	Cesarean section	Pfizer	Local pain
3	+	4	38	1	Cesarean section	Moderna	Local pain
4	+	2	33	2	Cesarean section	Pfizer	Local pain
5	+	25	31	1	Cesarean section	Pfizer	Local pain
6	+	3	36	2	Cesarean section	Pfizer	Local pain
7	+	2	33	2	Natural birth	Pfizer	Absent
8	+	33	31	1	Cesarean section	Pfizer	Generalized muscle pain, fever, headache
9	+	12	36	2	Cesarean section	Pfizer	Local pain
10	+	35	31	1	Natural birth	Pfizer	Local pain
11	+	19	35	1	Cesarean section	Pfizer	Absent
12	+	4	34	2	Natural birth	Pfizer	Local pain
13	+	10	32	1	Natural birth	Pfizer	Generalized muscle pain, fever, headache
14	+	10	32	1	Cesarean section	Pfizer	Absent
15	+	18	29	1	Natural birth	Pfizer	Absent
16	+	20	35	2	Cesarean section	Pfizer	Local pain
17	+	12	35	2	Cesarean section	Pfizer	Generalized muscle pain, fever, headache
18	+	2	37	2	Cesarean section	Moderna	Local pain
19	+	11	34	1	Cesarean section	Pfizer	Local pain
20	+	4	32	1	Cesarean section	Pfizer	Local pain
21	+	3	30	1	Cesarean section	Pfizer	Local pain
22	+	22	30	1	Natural birth	Pfizer	Local pain
23	+	15	35	1	Natural birth	Pfizer	Absent
24	+	12	36	2	Cesarean section	Moderna	Local pain
25	+	17	34	1	Cesarean section	Pfizer	Local pain
26	+	34	32	1	Cesarean section	Pfizer	Generalized muscle pain, fever, headache

Table S2. Characteristics of SARS – CoV – 2 infected mothers group

ID No	Anti-RBD IgG	Child`s age (months)	Mother`s age (years)	Mother`s parity	Birth type	Vaccine prior infection	Symptoms	Hospita-lization	Oxygen therapy
1	+	4	37	2	Cesarean section	Yes	sore throat, asthenia	No	No
2	+	6	35	1	Cesarean section	Yes	significant asthenia, altered general condition, cough, sore throat	No	No
3	+	2	31	2	Natural birth	No	sore throat, fever, muscle pain	No	No
4	+	7	30	2	Cesarean section	Yes	sore throat, lack of smell/taste	No	No
5	+	3	32	2	Cesarean section	No	fever, muscle pain	No	No
6	+	11	30	1	Natural birth	Yes	significant asthenia, altered general condition, fever, cough, sore throat	No	No
7	+	6	36	3	Cesarean section	Yes	significant asthenia, altered general condition, fever	No	No
8	+	28	31	1	Cesarean section	Yes	lack of smell/taste	No	No
9	+	21	33	2	Natural birth	No	sore throat, muscle pain	No	No
10	+	16	35	2	Natural birth	Yes	lack of smell/taste	No	No
11	+	33	31	1	Natural birth	Yes	lack of smell/taste, fever, muscle pain	No	No
12	+	24	32	1	Cesarean section	No	significant asthenia, altered general condition, cough, sore throat, muscle pain	No	No
13	+	14	38	3	Natural birth	Yes	significant asthenia, altered general condition, cough, fever, muscle pain	No	No
14	+	9	32	1	Cesarean section	Yes	significant asthenia, altered general condition, cough, fever	No	No
15	+	20	36	2	Cesarean section	Yes	sore throat, muscle pain	No	No
16	+	13	29	2	Cesarean section	No	sore throat, fever	No	

17	+	21	28	2	Natural birth	No	significant asthenia, altered general condition, cough, fever	No	
18	+	13	32	2	Cesarean section	Yes	significant asthenia, altered general condition, cough, fever	No	
19	+	16	38	2	Natural birth	Yes	sore throat, fever, muscle pain	No	
20	+	4	36	2	Cesarean section	Yes	sore throat, lack of smell/taste	No	
21	+	7	32	1	Cesarean section	Yes	significant asthenia, altered general condition, fever, muscle pain	No	
22	+	8	31	1	Cesarean section	Yes	sore throat, fever, lack of smell/taste	No	

Table S3. Characteristics of the control group

**ID No**	**Anti-RBD IgG**	**Child`s age (months)**	**Mother`s age (years)**	**Mother`s parity**	**Birth type**
1	-	9	32	2	Natural birth
2	-	13	29	2	Cesarean section
3	-	24	33	2	Natural birth
4	-	9	34	2	Cesarean section
5	-	12	30	2	Cesarean section
6	-	5	31	1	Natural birth
7	-	2	33	3	Cesarean section
8	-	16	33	1	Natural birth
9	-	3	36	2	Natural birth
10	-	7	30	2	Natural birth
11	+	8	36	2	Cesarean section
12	+	26	35	2	Cesarean section
13	+	2	32	1	Natural birth
14	+	2	31	2	Natural birth
15	+	19	29	3	Natural birth
16	+	11	44	3	Natural birth
17	+	8	31	1	Natural birth

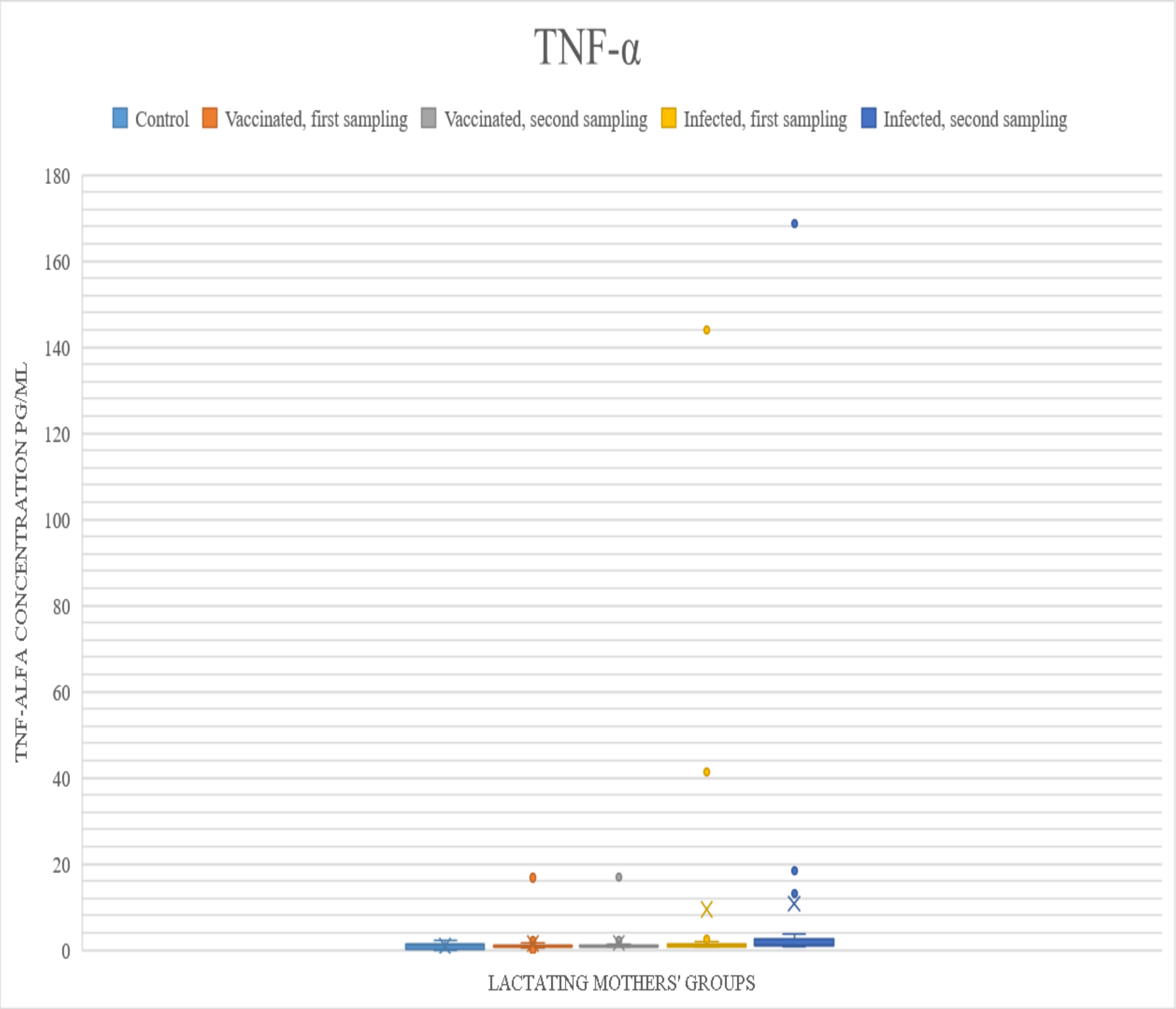

Figure S1: TNF-α concentrations range according to each study group

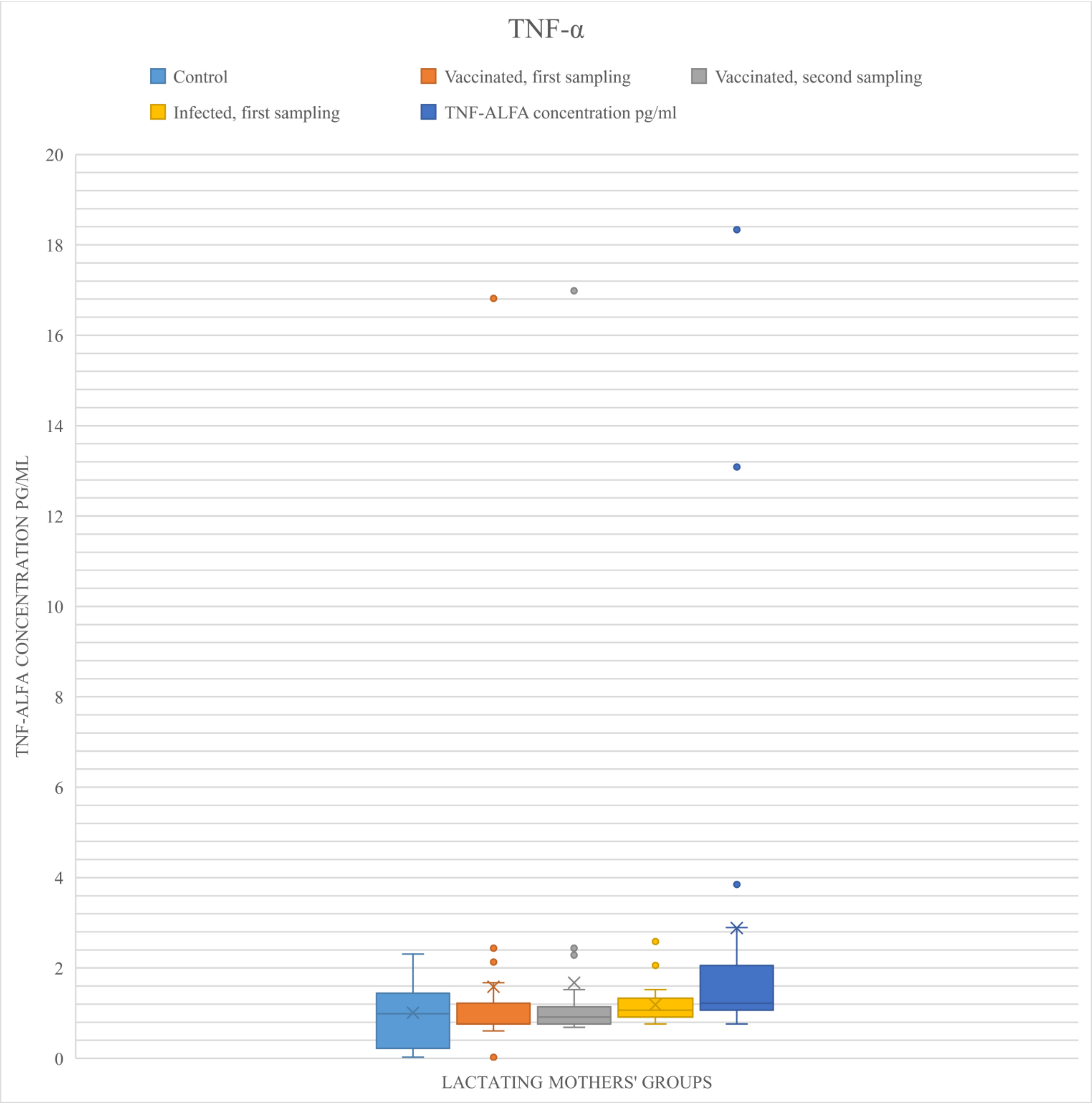

Figure S2: TNF-α concentrations range according to each study group, without extreme outliners for a better visibility

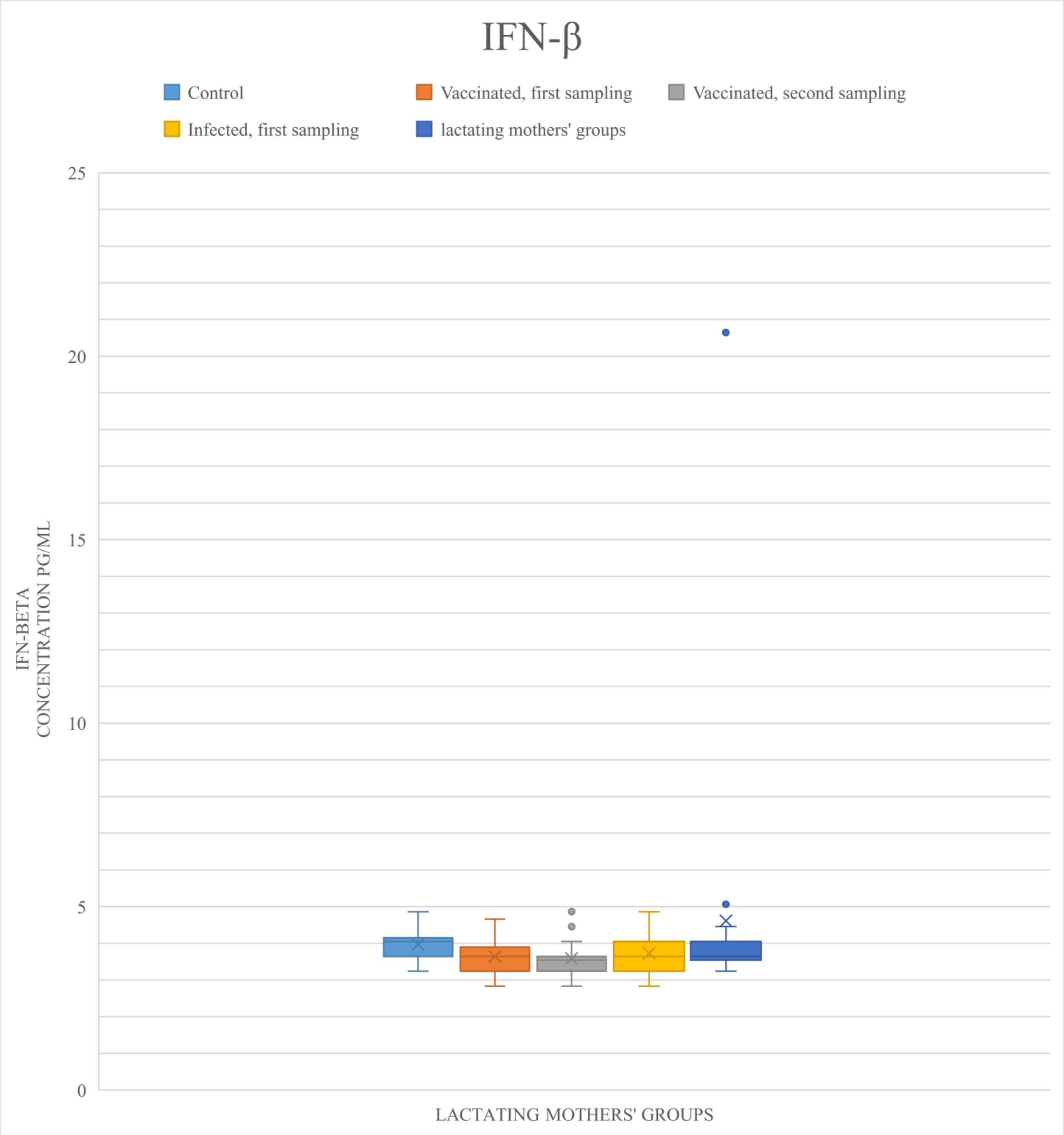

Figure S3: IFN-β concentrations range according to each study group

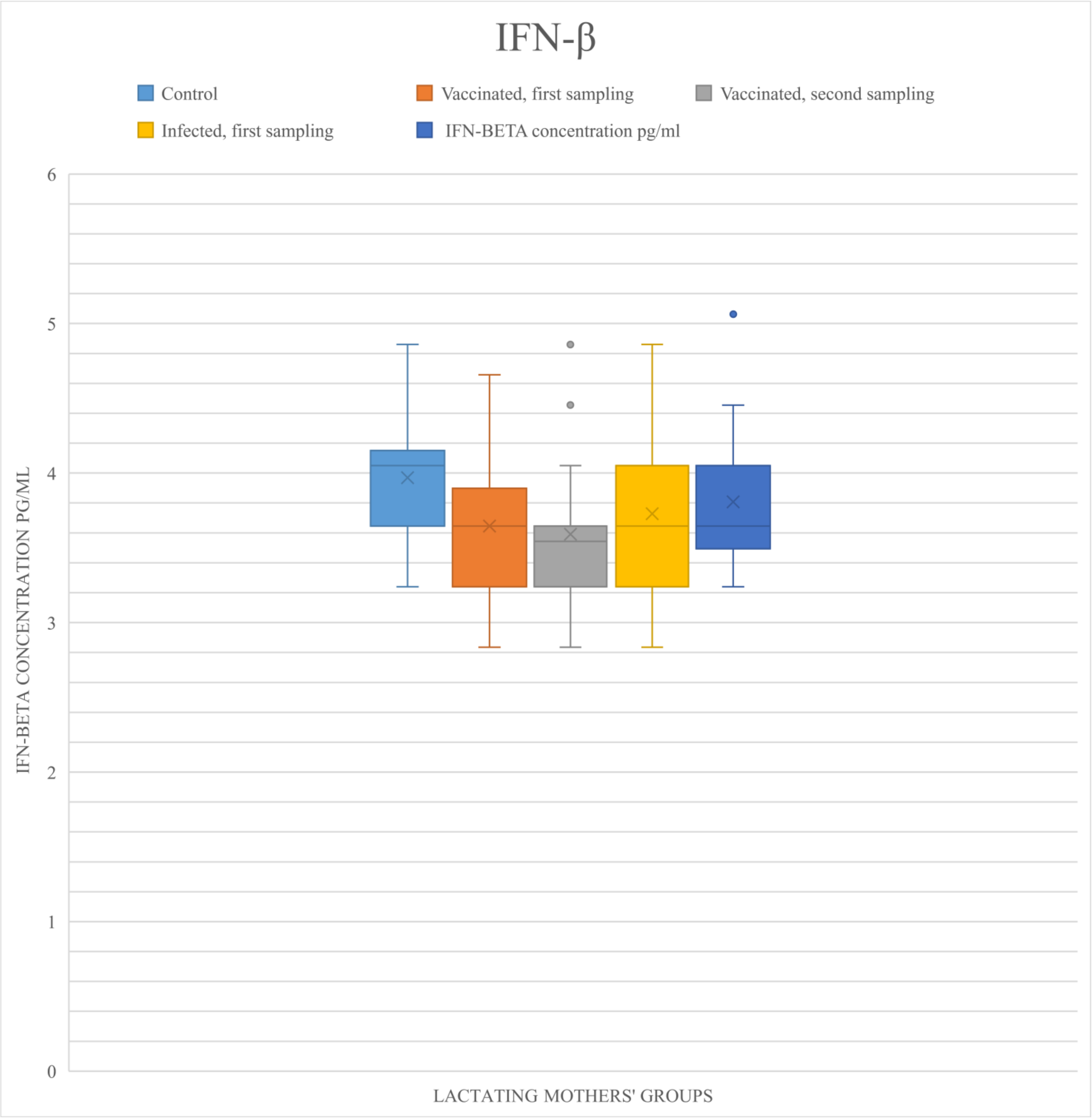

Figure S4: IFN-β concentrations range according to each study group, without extreme outliners for a better visibility

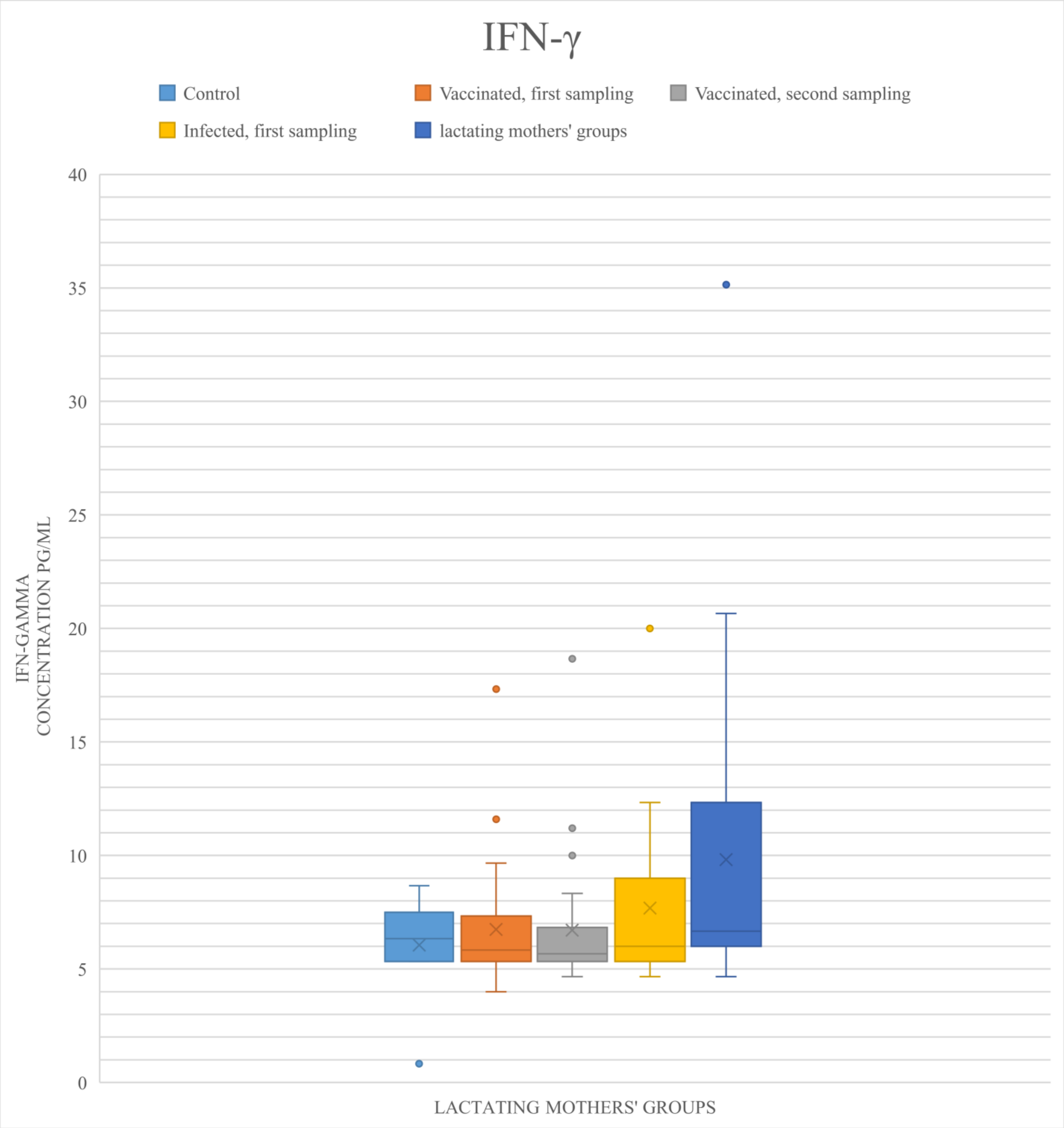

Figure S5: IFN-γ concentrations range according to each study group

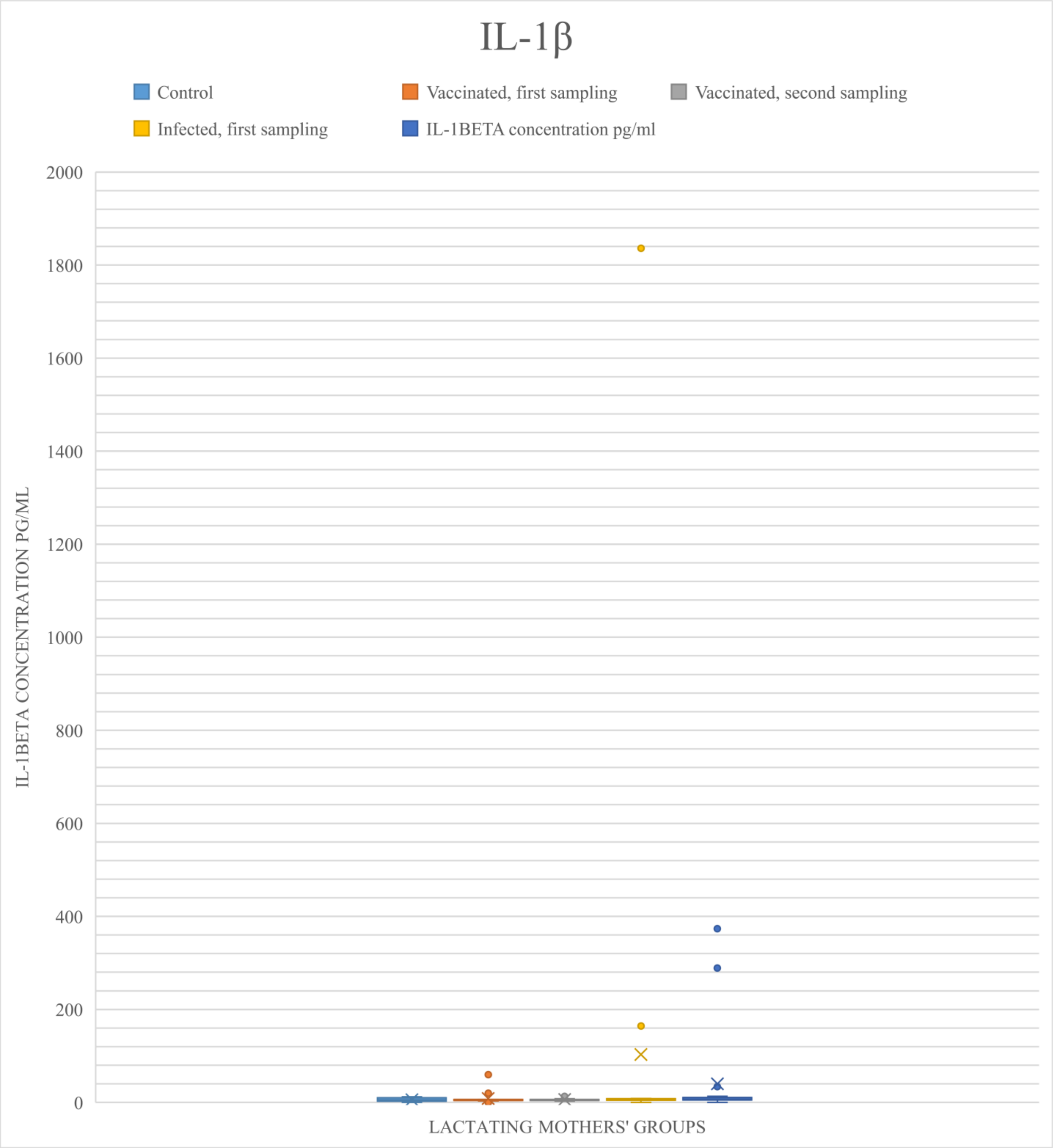

Figure S6: IL-1β concentrations range according to each study group

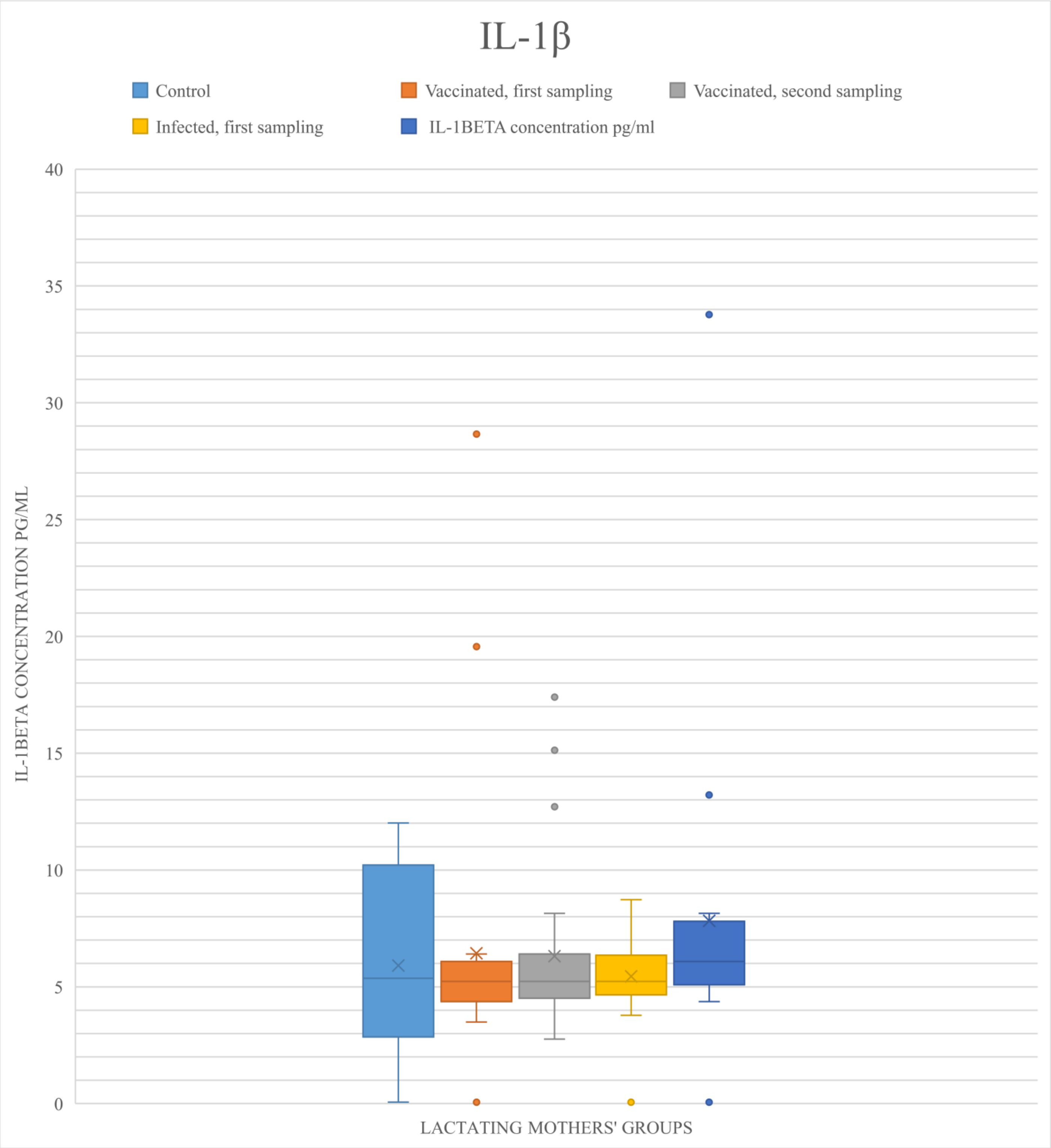

Figure S7: IL-1β concentrations range according to each study group, without extreme outliners for a better visibility

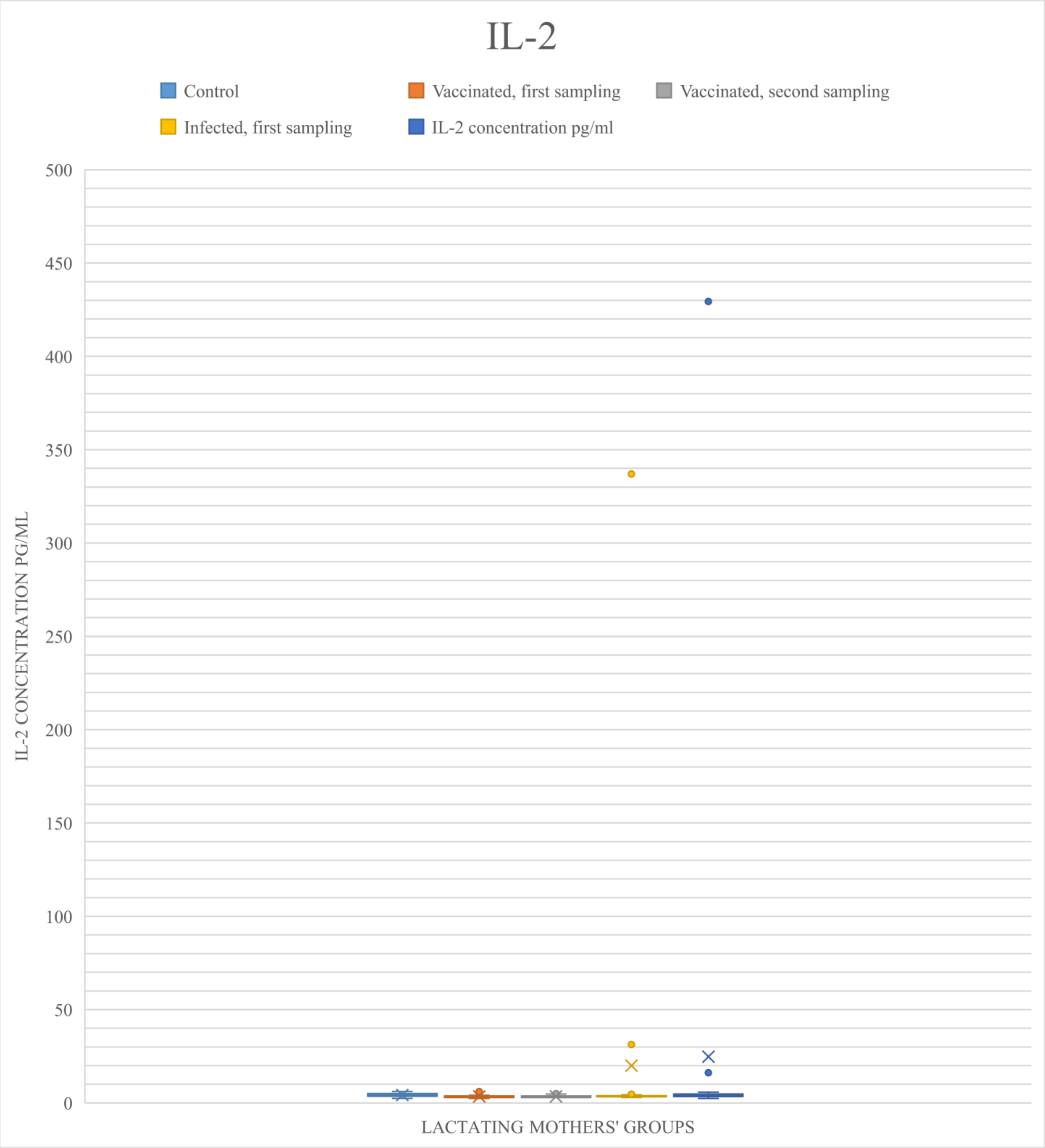

Figure S8: IL-2 concentrations range according to each study group

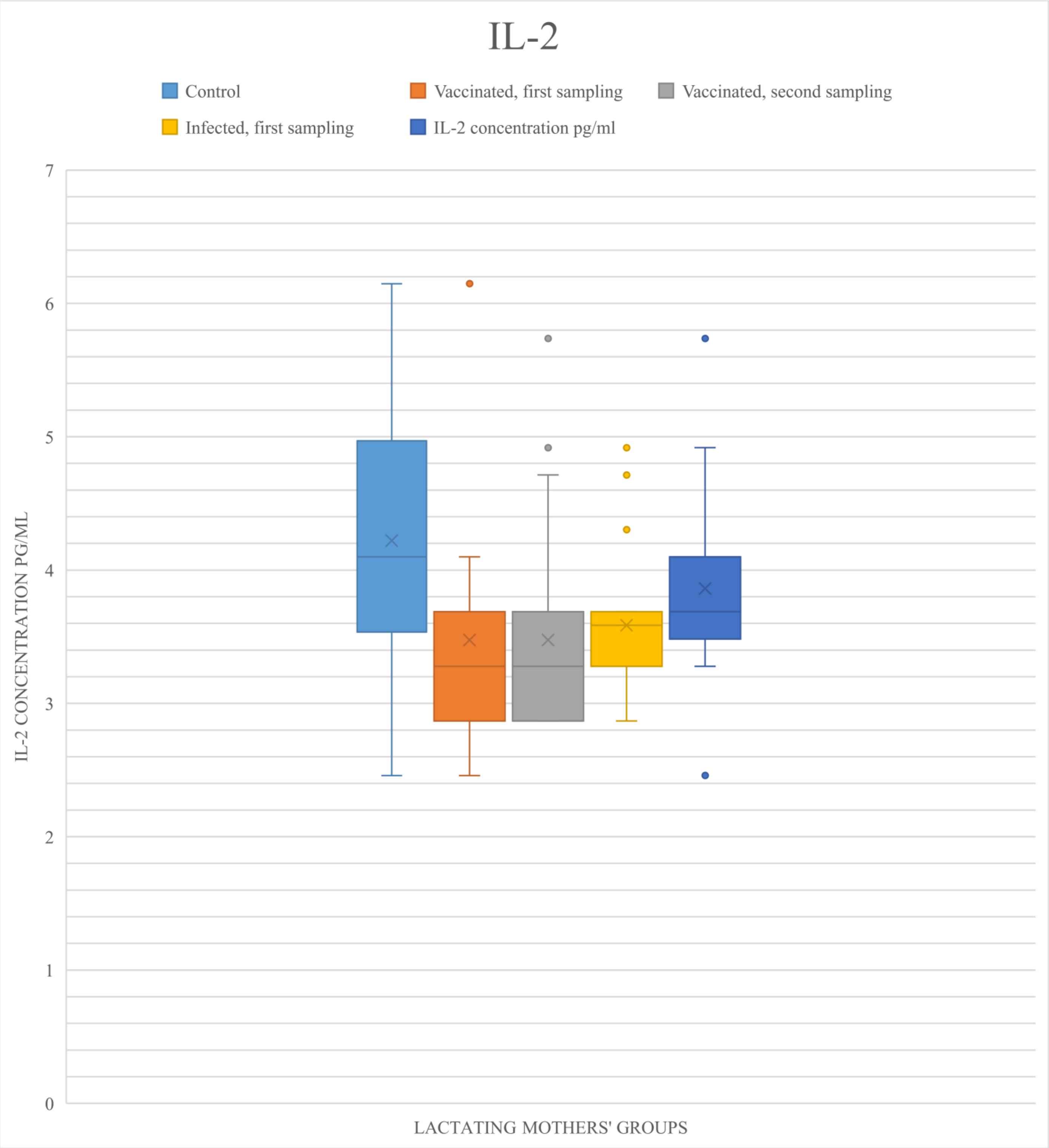

Figure S9: IL-2 concentrations range according to each study group, without extreme outliners for a better visibility

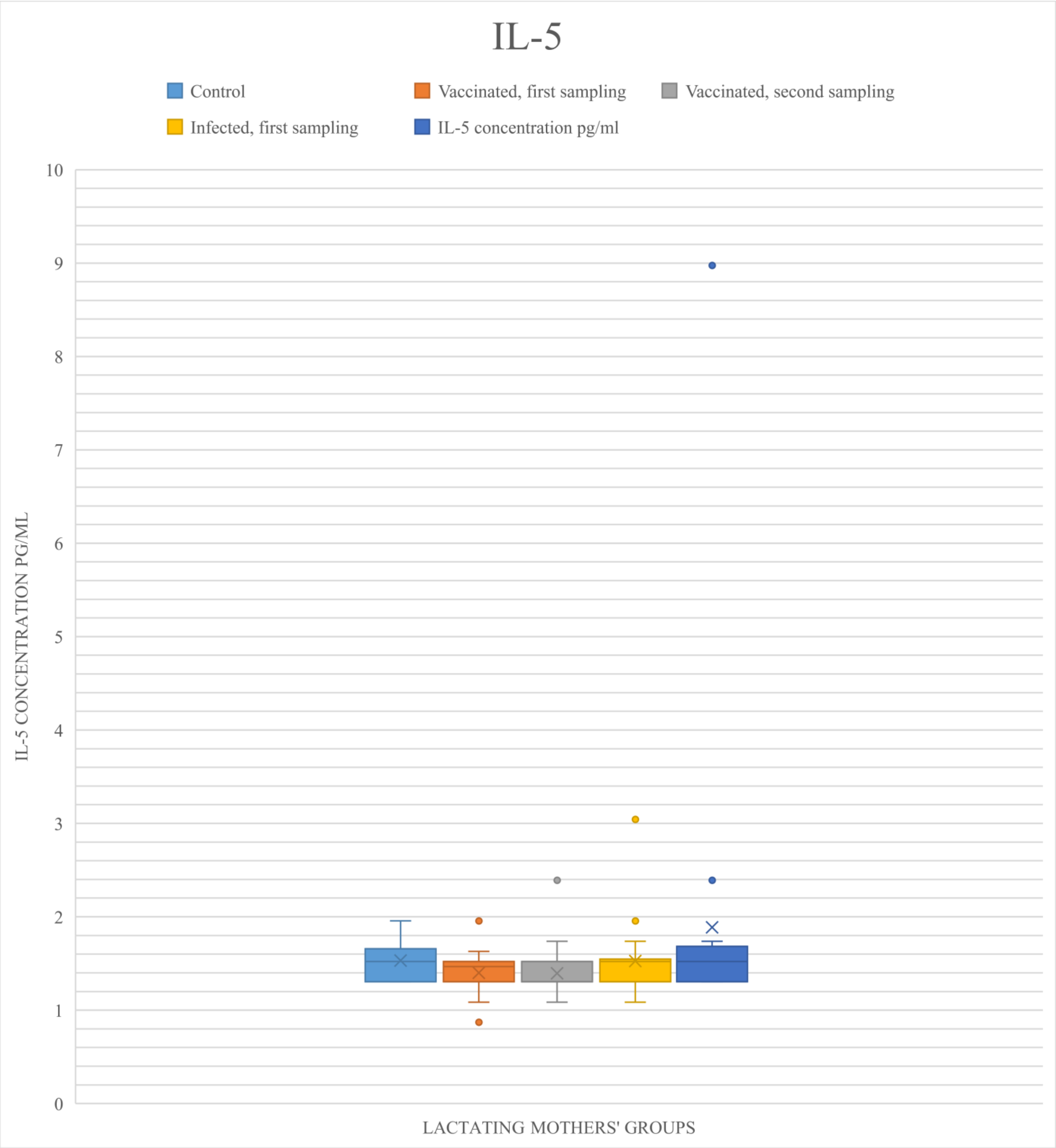

Figure S10: IL-5 concentrations range according to each study group

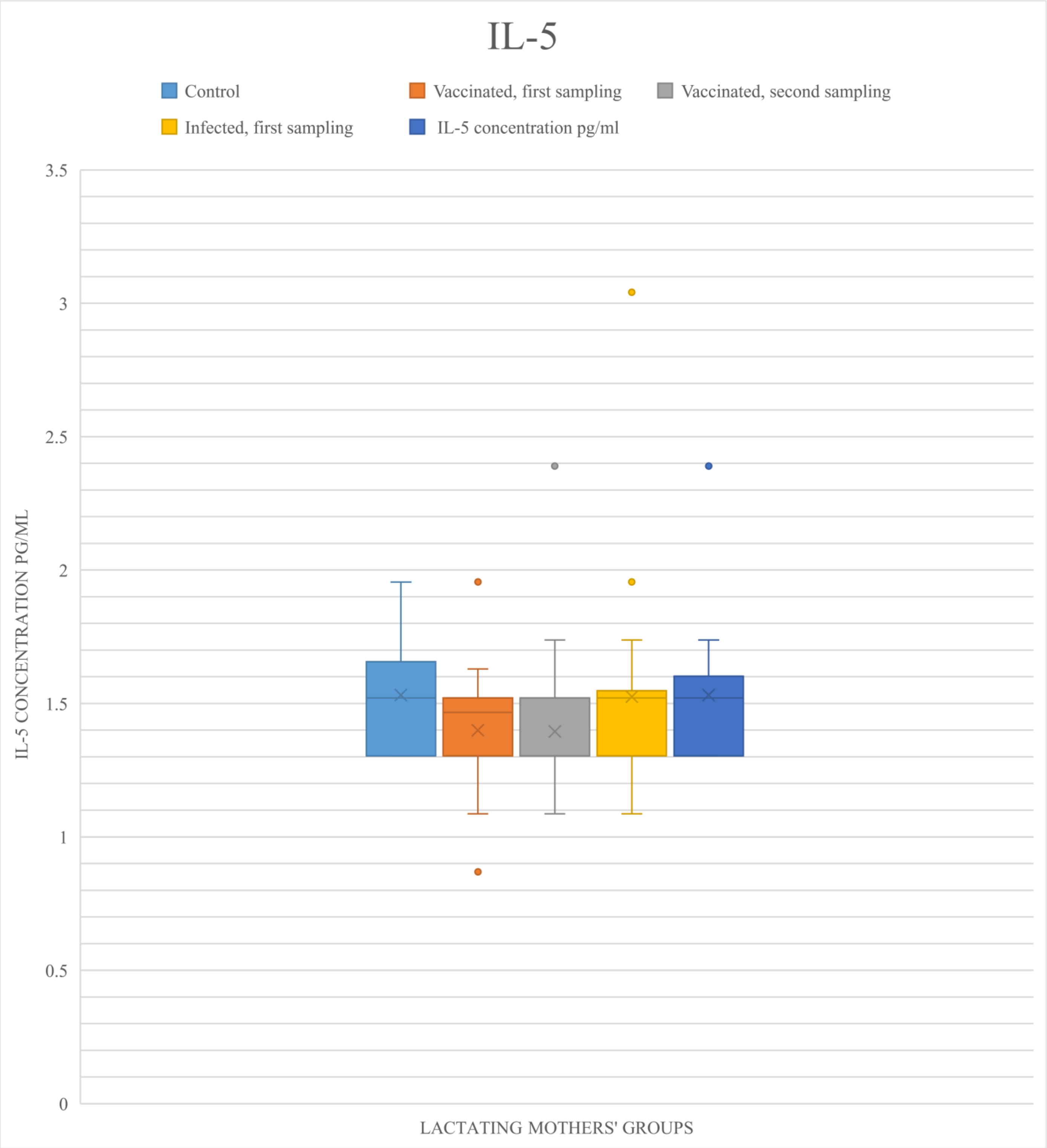

Figure S11: IL-5 concentrations range according to each study group, without extreme outliners for a better visibility

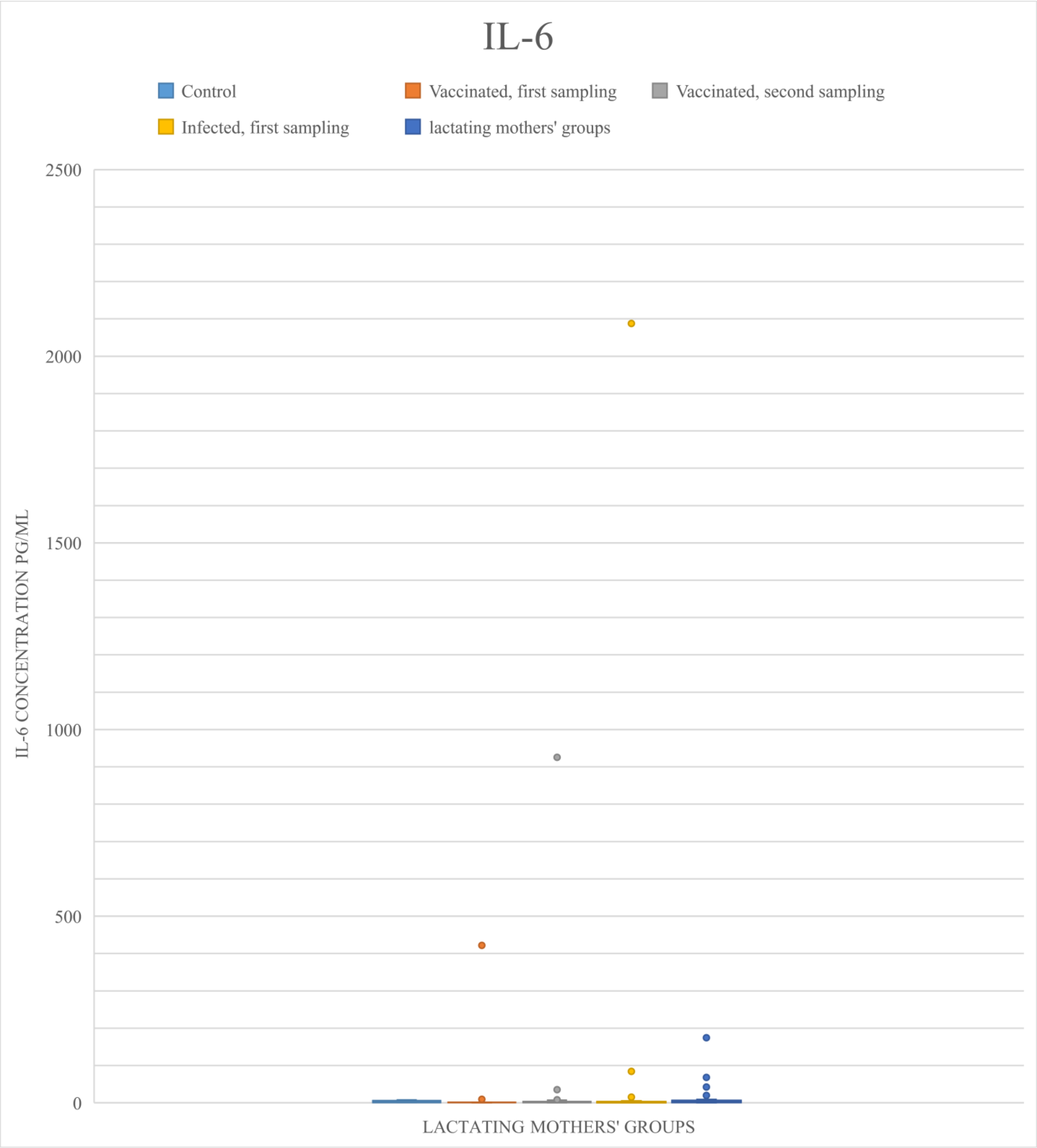

Figure S12: IL-6 concentrations range according to each study group

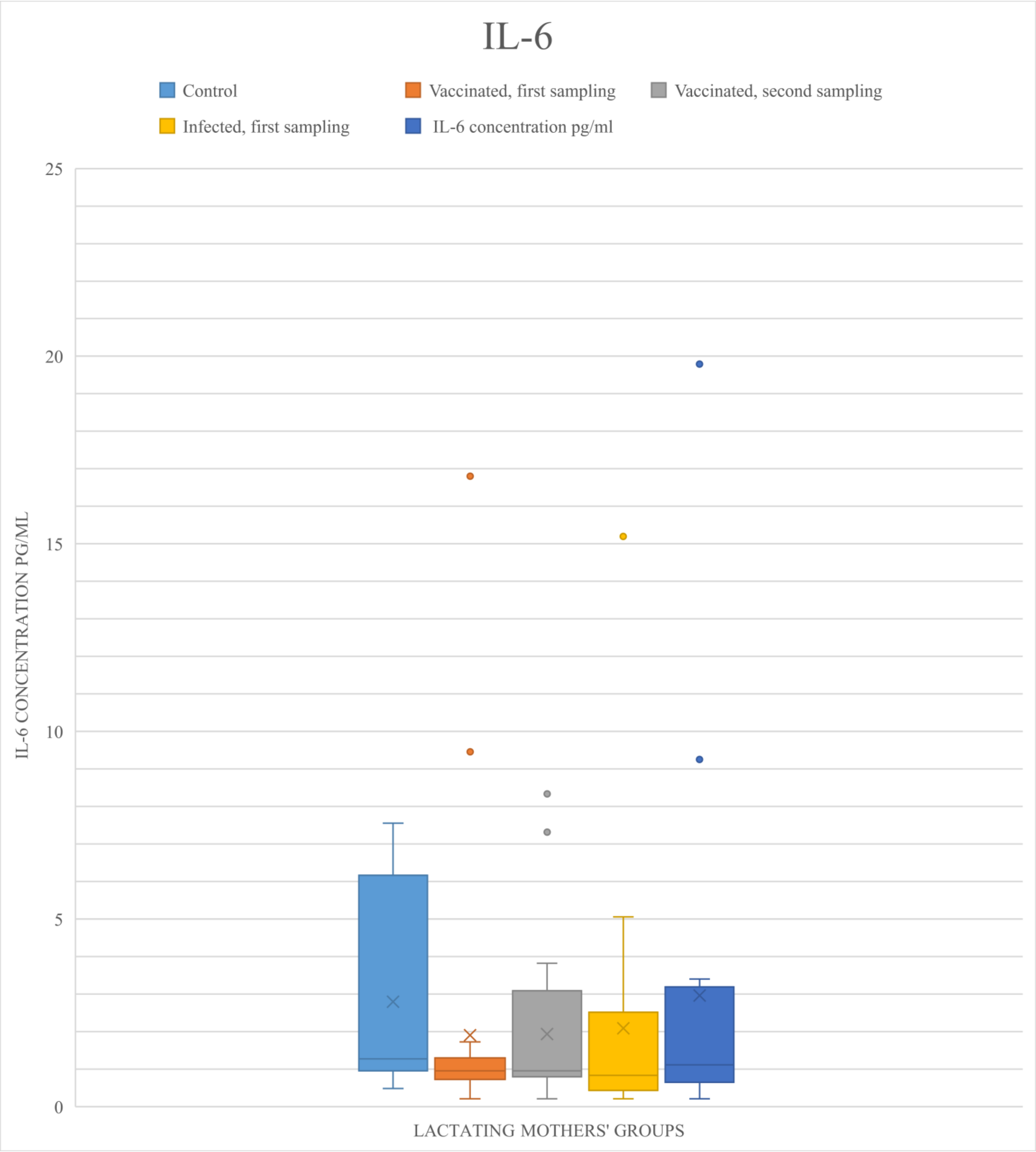

Figure S13: IL-6 concentrations range according to each study group, without extreme outliners for a better visibility

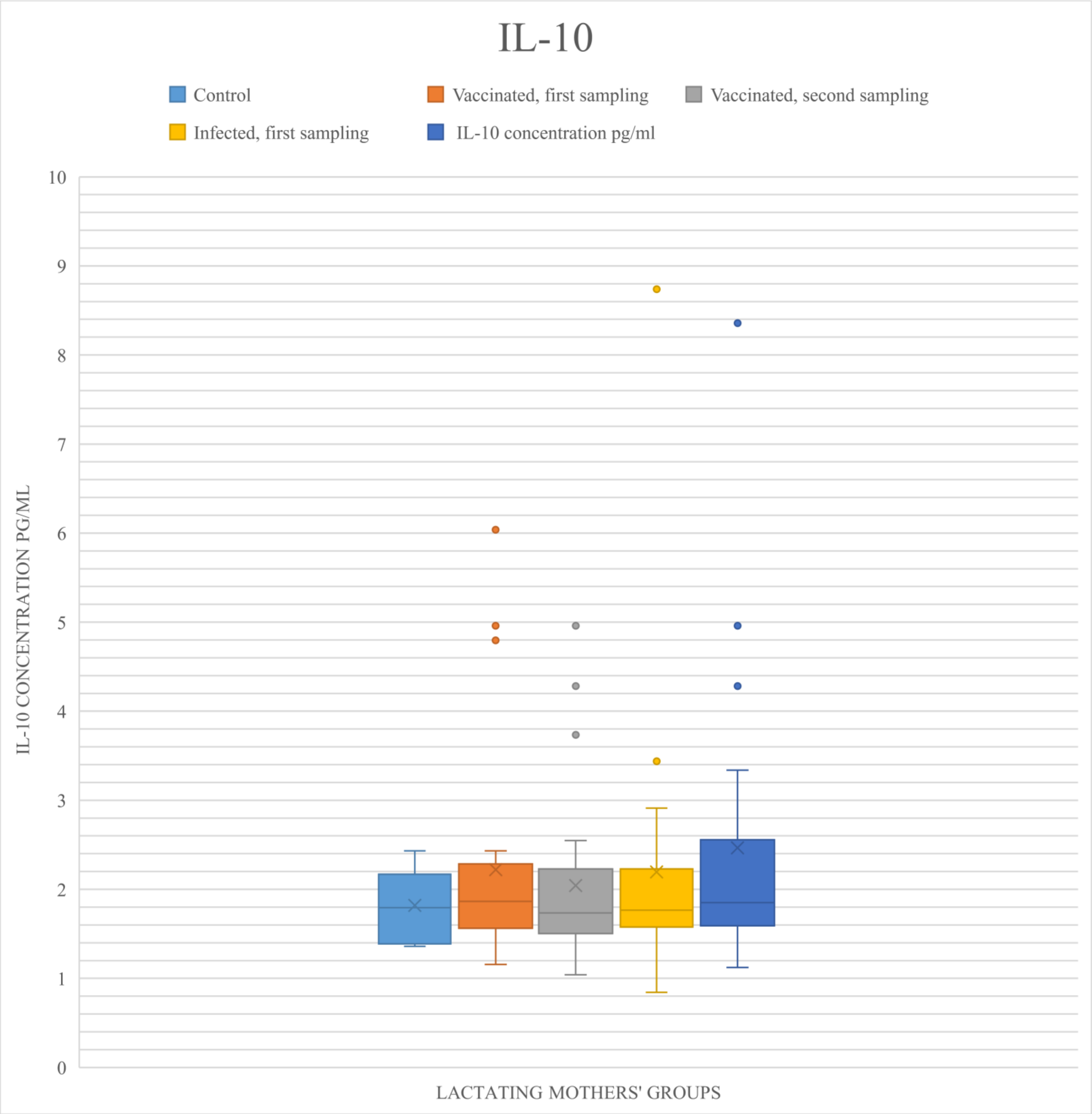

Figure S14: IL-10 concentrations range according to each study group

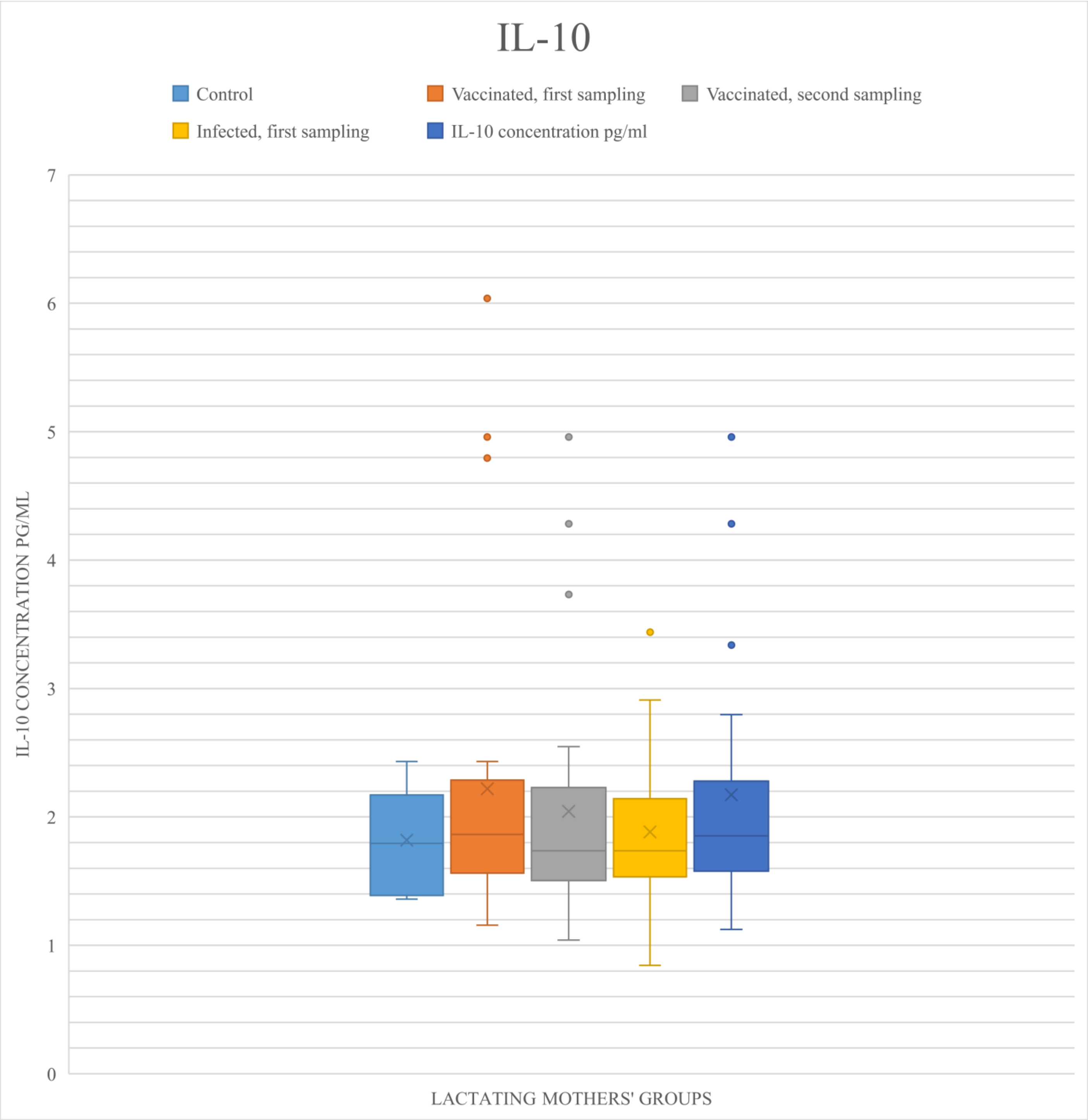

Figure S15: IL-10 concentrations range according to each study group, without extreme outliners for a better visibility

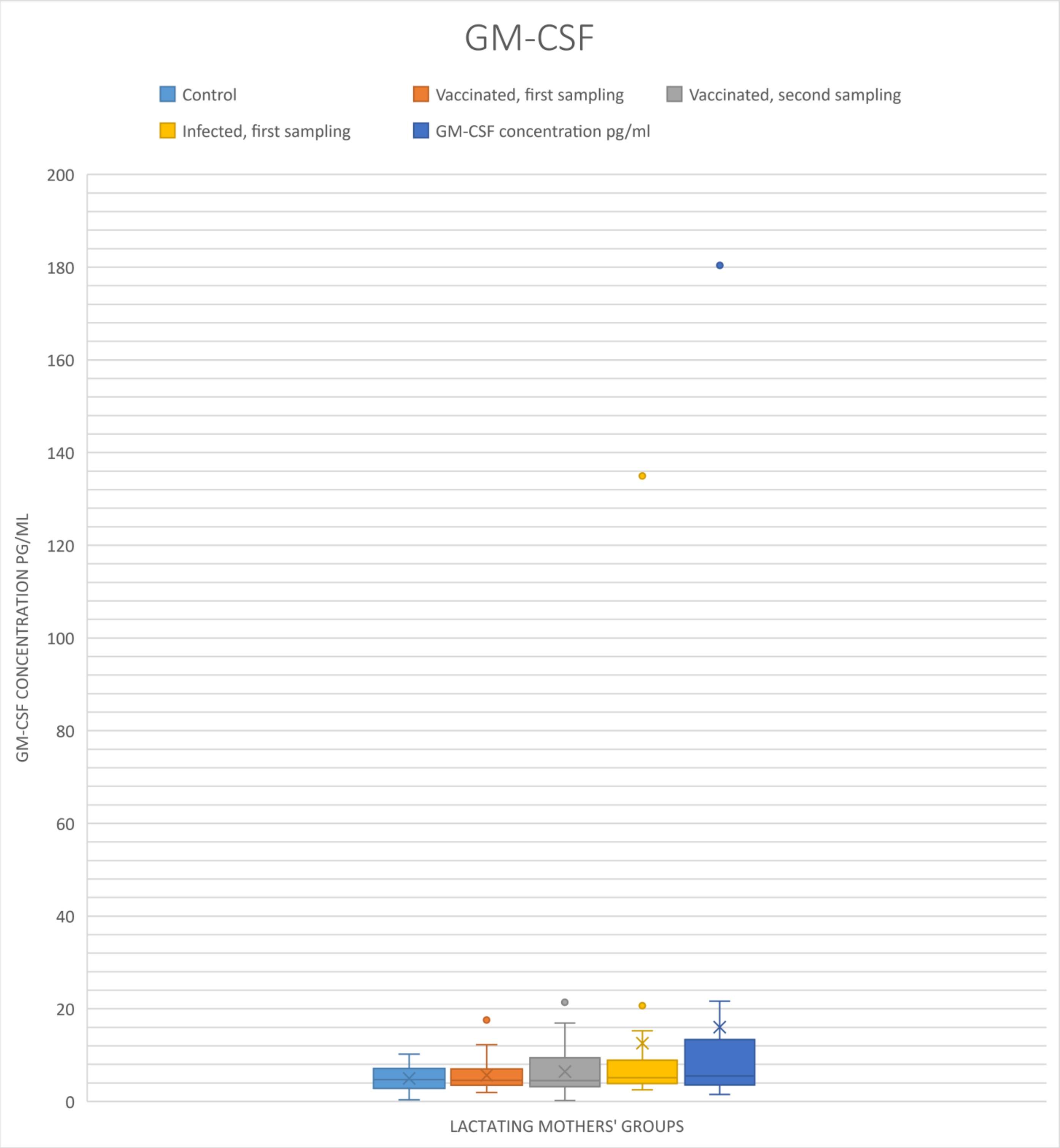

Figure S16: GM-CSF concentrations range according to each study group

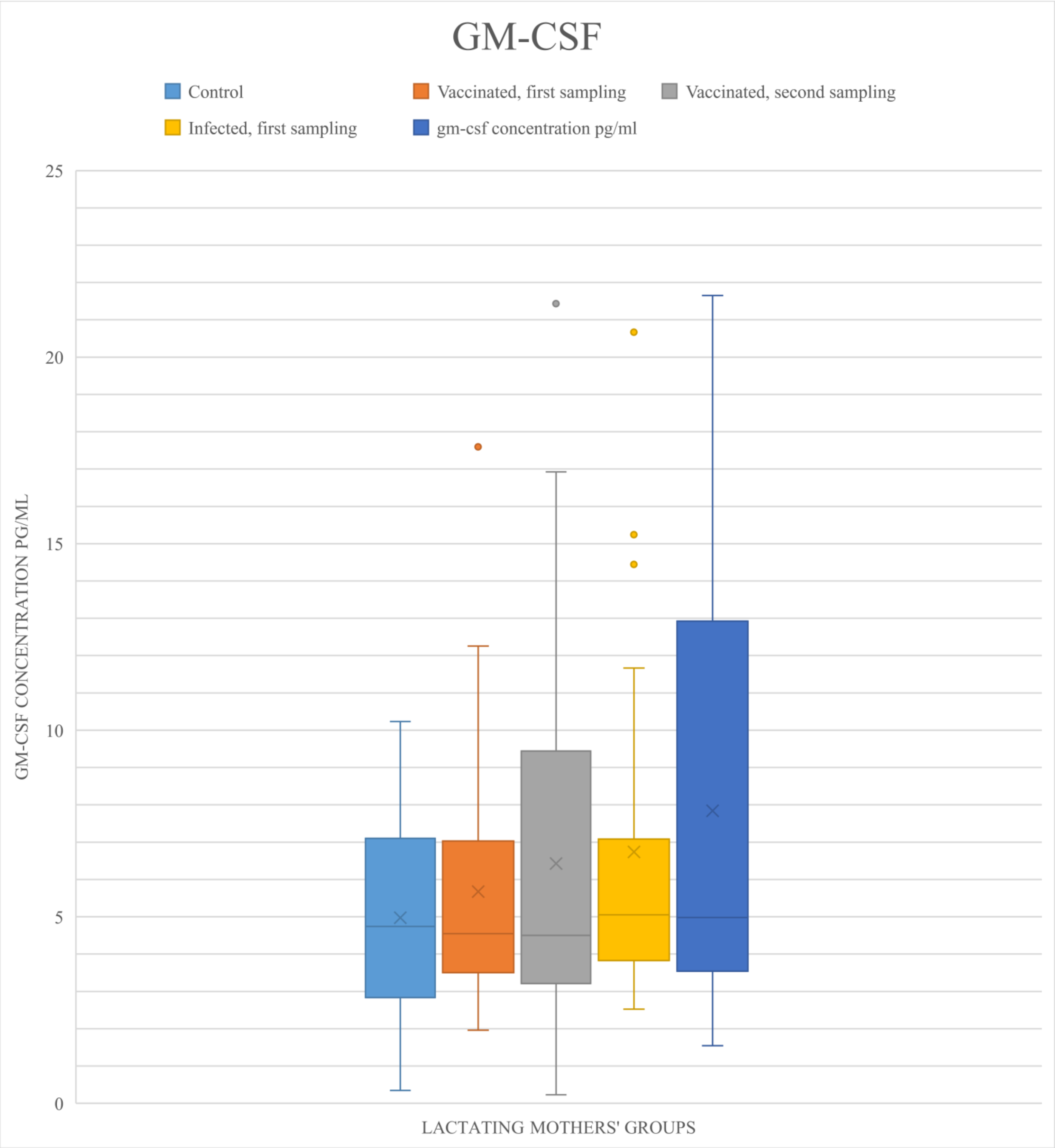

Figure S17: GM-CSF concentrations range according to each study group, without extreme outliners for a better visibility

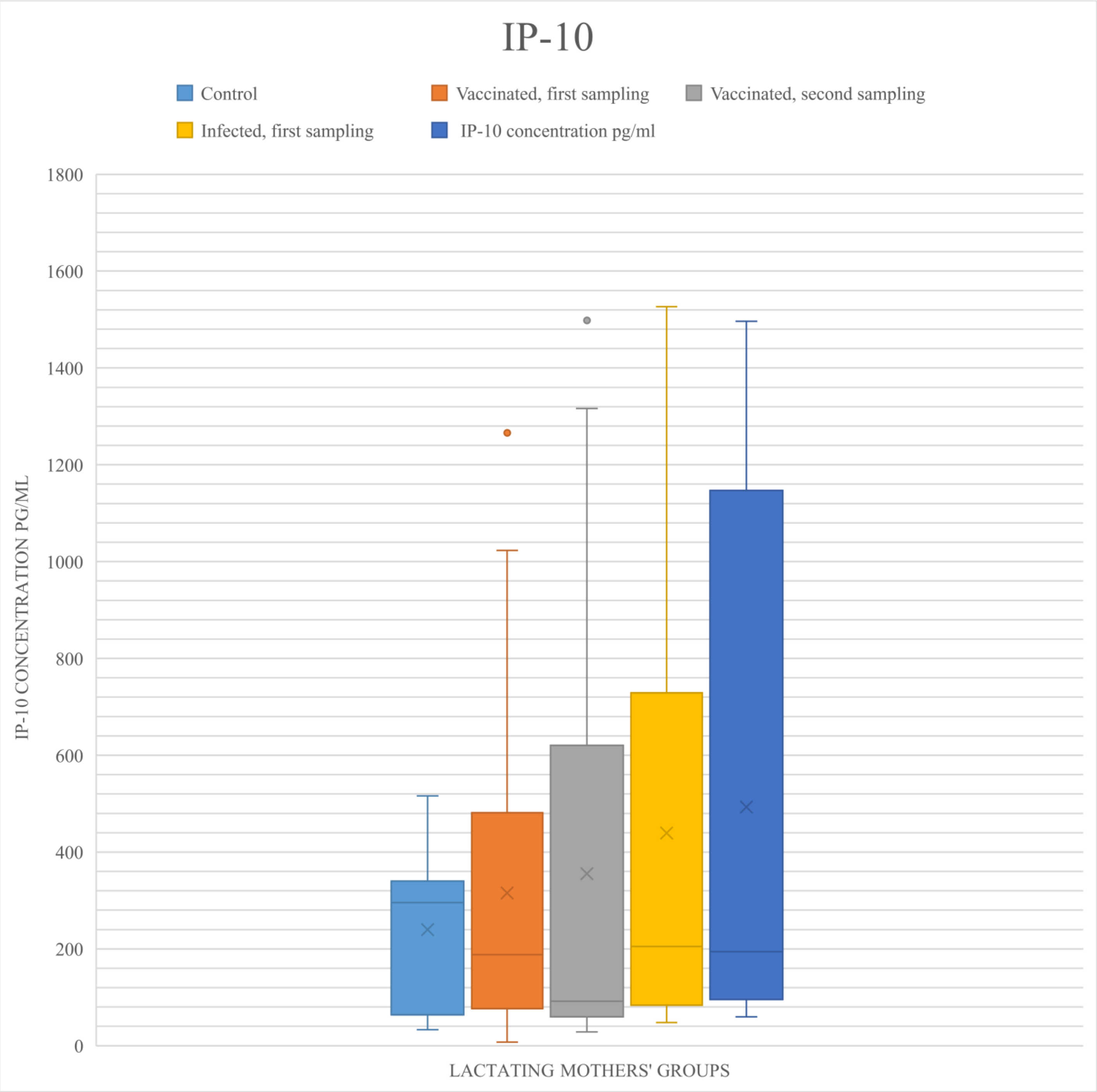

Figure S18: IP-10 concentrations range according to each study group

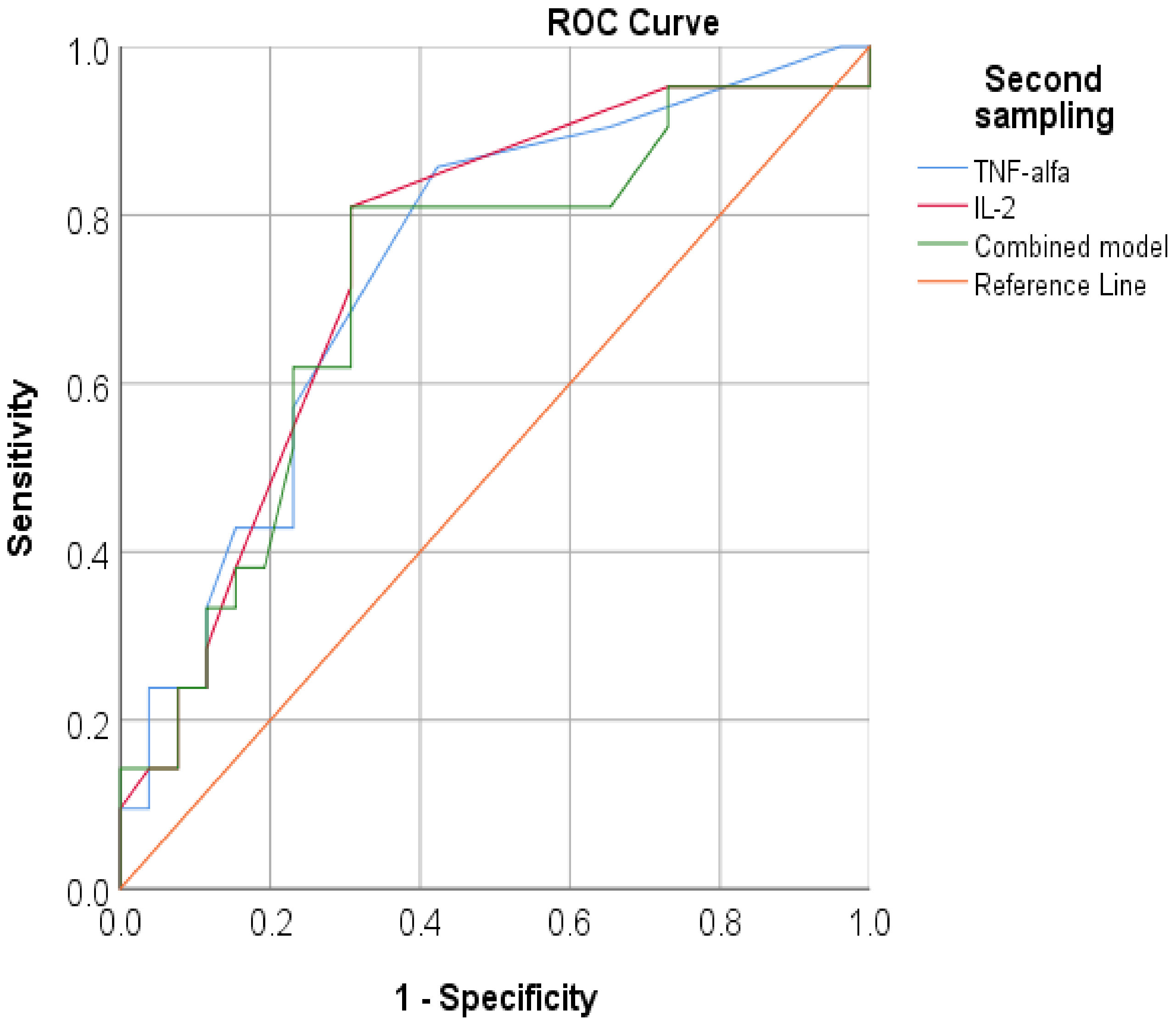

Figure S19: ROC curves generated for biomarkers that best predicted the infection status: TNF-alpha and IL-2. *TNF-alpha and IL-2 equally predicted the infection status in our study group. The last model comprises the combined effect of these two selected biomarkers. For this analysis, the larger values of the test variables suggest indicate stronger evidence of a positive actual state (SARS-CoV-2 infection), while the AUC values between 0.7-0.8 define a good capacity of discrimination.*
